# Pervaporation Mixed Matrix Membranes from Sodium Alginate/ZnO for Isopropanol Dehydration

**DOI:** 10.3390/molecules31081300

**Published:** 2026-04-16

**Authors:** Roman Dubovenko, Mariia Dmitrenko, Anna Mikulan, Olga Mikhailovskaya, Anna Kuzminova, Aleksandra Koroleva, Anton Mazur, Rongxin Su, Anastasia Penkova

**Affiliations:** 1 St. Petersburg State University, 7/9 Universitetskaya nab., St. Petersburg 199034, Russia; r.dubovenko@spbu.ru (R.D.); st097675@student.spbu.ru (A.M.); st113220@student.spbu.ru (O.M.); a.kuzminova@spbu.ru (A.K.); aleksandra.koroleva@spbu.ru (A.K.); a.mazur@spbu.ru (A.M.); 2 State Key Laboratory of Chemical Engineering, School of Chemical Engineering and Technology, Tianjin University, Tianjin 300072, China; surx@tju.edu.cn

**Keywords:** sodium alginate, metal oxide, ZnO, pervaporation, isopropanol dehydration

## Abstract

In this work, sodium alginate (NaAlg) membranes were enhanced with synthesized zinc oxide (ZnO) nanoplates to enable efficient pervaporation dehydration of isopropyl alcohol (IPA). A comprehensive suite of characterisation techniques—scanning electron (SEM) and atomic force (AFM) microscopy, Fourier-transform infrared (FTIR) spectroscopy, nuclear magnetic resonance (NMR), X-ray diffraction (XRD), X-ray photoelectron spectroscopy (XPS), thermogravimetric analysis (TGA), contact angle and liquid uptake measurements—along with density functional theory (DFT) calculations, was employed to establish robust structure–property relationships and to elucidate filler–polymer interactions. Membranes with different ZnO contents were prepared, and membranes based on the optimal NaAlg-ZnO(5%) composite were cross-linked with CaCl_2_ to improve stability in aqueous solutions, and supported membranes were developed for prospective applications by applying this composite onto the prepared porous cellulose acetate (CA) substrate. This developed cross-linked supported NaAlg-ZnO(5%)/CA membrane had a permeation flux increased by 2 times or more compared to a dense NaAlg membrane during dehydration of IPA (12–30 wt.% water) with a permeate water content above 99 wt.%. The integrated experimental–theoretical approach provides mechanistic insight into ZnO–NaAlg interactions and demonstrates the strong potential of these mixed matrix membranes for high-efficiency alcohol dehydration, offering a rational design paradigm for next-generation pervaporation membranes.

## 1. Introduction

Over the past decade, membrane technologies have gained widespread application in water purification (ultrafiltration, nanofiltration, and reverse osmosis) [1], gas separation [2], and the dehydration of organic solvents, particularly alcohols [3]. The dehydration process attracts considerable attention due to its significant advantages over azeotropic distillation: it enables a reduction in high energy consumption, utilizes simple, compact, and modular equipment, does not require the use of toxic substances, and ensures the production of a high-purity product. Specifically, recent work has demonstrated that the pervaporation method can reduce the cost of producing high-purity isopropyl alcohol (IPA) by up to half [4]. Concurrently, the rapid development of the chemical, pharmaceutical, and electronics industries is driving increasing demands for the purity of alcohol solvents, which is essential for ensuring the high quality of value-added products [5,6,7]. The efficiency of the pervaporation process is highly dependent on the membrane material used, driving the demand for new materials with enhanced transport properties [8].

In line with the global drive to shift from synthetic petroleum-based polymers to natural biopolymers, particularly polysaccharides and their derivatives, a number of studies have been published demonstrating their competitive performance compared to petroleum-based polymers [9,10,11,12]. Sodium alginate (NaAlg), widely used for creating hydrogels, has been proven effective for hydrophilic pervaporation (water separation) [7,13]. This is due to its pronounced hydrophilicity with a high density of carboxyl and hydroxy groups, excellent water selectivity, film-forming ability, biodegradability, and availability [14,15,16]. Its primary limitations, however, remain solubility in mixtures with high water content, excessive swelling leading to non-selective transport, and the characteristic polymer trade-off between selectivity and permeability [8].

To overcome these limitations, the strategy of creating mixed matrix membranes (MMMs) through bulk modification of the polymer matrix has gained broad acceptance [15,17,18,19]. The main challenge is centered on selecting a suitable polymer–modifier pair, as excessive interaction can bind polymer chains, reducing performance, while poor affinity between the polymer and filler can lead to the formation of non-selective voids [20]. Moreover, studies indicate that introducing modifiers significantly affects the morphology, free volume, degree of crystallinity, and surface properties of MMMs [21]. Over the last decade, researchers have proposed various types of modifiers: carbon nanoparticles [9,22], metal–organic frameworks [7], covalent organic frameworks [23], zeolites [24], and metal oxides [25], which can substantially improve transport characteristics. Among metal oxides, TiO_2_, ZnO, CuO, Al_2_O_3_, Fe_3_O_4_, and Cr_2_O_3_ have found the widest use as nanofillers due to the ability to tune their characteristics (size, shape, etc.) during synthesis, as well as their relative ease of production [13,25,26]. It should be noted that zinc oxide particles possess physicochemical properties similar to those of TiO_2_ and, being approximately four times cheaper, represent a cost-effective alternative to this well-established filler, although they have been less extensively studied [27]. The advantages of ZnO include its chemical and thermal stability, good biocompatibility, low toxicity, bactericidal and antimicrobial properties [28,29]. An important feature is also the high hydrophilicity of ZnO and its affinity for polymer’s hydrophilic functional groups such as –OH, –SO_3_H, and –COOH, due to the formation of non-covalent interactions [28]. Despite the popularity of ZnO as a modifier for creating porous membranes, examples of its application in MMMs for pervaporation remain limited.

Thus, in the work of M. Şahin et al., the introduction of 15 wt.% ZnO into a polyvinyl alcohol matrix for dehydrating a 20 wt.% acetone/water mixture at 40 °C increased the permeate flux by 2.5 times (to 0.2 kg m^−2^h^−1^) [30]. In another study by Y. Wang et al., the incorporation of ZnO into a cellulose acetate matrix during the separation of a methanol/methyl tert-butyl ether mixture led to a 96.5% increase in flux and a 48% increase in selectivity [26]. Continuing this research for the separation of a 71 wt.% IPA/water mixture, it was shown that the additional introduction of 10 wt.% polyethylene glycol 600 makes it possible to achieve a high selectivity of 1448 with a permeation flux of 0.559 kg m^−2^h^−1^ for IPA dehydration [31]. Thus, the potential of using ZnO as a modifier has been demonstrated; however, to the best of our knowledge, no examples of a systematic investigation on the effect of incorporating ZnO into a NaAlg polymer matrix for creating pervaporation membranes for dehydrating water/IPA mixtures have been reported in the scientific literature.

Thus, the aim of this work was to develop and investigate pervaporation MMMs based on NaAlg modified with synthesized ZnO nanoplates for enhanced dehydration of IPA, as well as to establish the structure-property relationship. ZnO nano-objects were synthesized and characterized, dense and supported membranes were fabricated, and the influence of the filler and cross-linking on the structural characteristics, morphology, thermal stability, hydrophilicity, and surface topography of the membranes was systematically investigated. A wide range of analytical methods was employed for this purpose, including FTIR, XRD, XPS, NMR, TGA, SEM, AFM, low-temperature nitrogen adsorption, contact angle and liquid uptake measurements. Furthermore, a computational study was conducted to investigate non-covalent interactions within the system. The final stage involved testing the fabricated membranes in the process of pervaporation dehydration of water/IPA mixtures across a wide concentration range. To the best of our knowledge, this study represents the first systematic work on the application of ZnO nanoplates as a modifier for NaAlg-based membranes for pervaporation purposes.

## 2. Results and Discussion

### 2.1. ZnO Investigation

**Figure 1:**
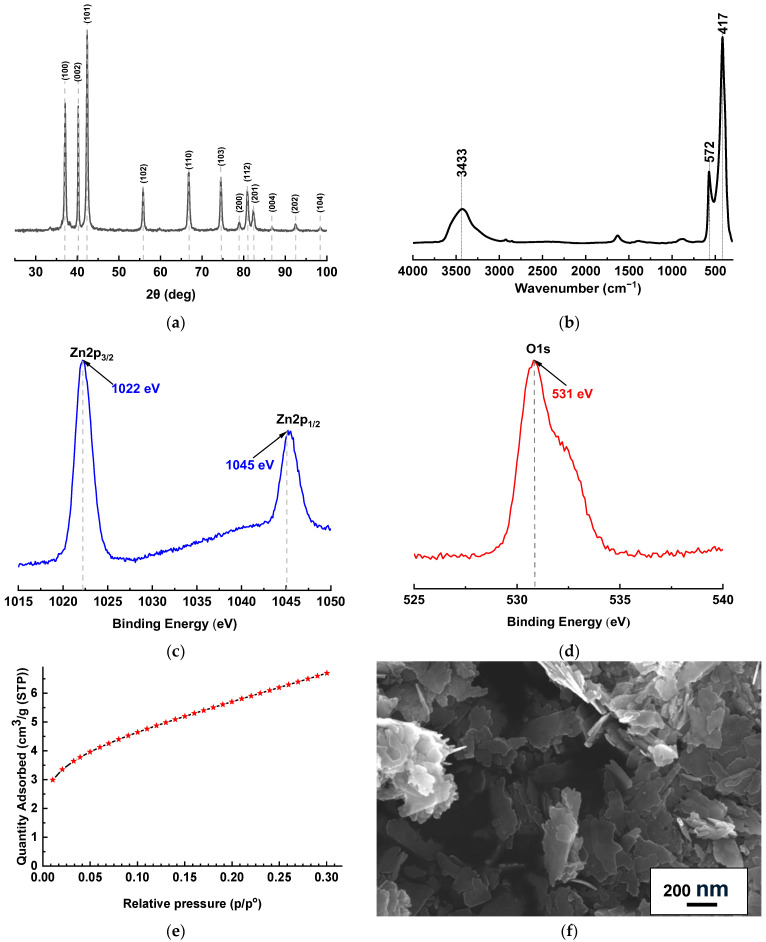
(**a**) XRD, (**b**) FTIR, and (**c**,**d**) high-resolution XPS spectra, (**e**) N_2_ adsorption isotherm, and (**f**) SEM micrograph of the synthesized ZnO.

The phase composition and crystal structure of the synthesized ZnO were investigated by XRD (Figure 1a). The diffraction pattern exhibits well-defined peaks at 2θ~37.1°, 40.2°, 42.3°, 55.8°, 66.8°, 74.5°, 78.9°, 80.9°, and 82.3°, corresponding to the (100), (002), (101), (102), (110), (103), (200), (112), and (201) planes of hexagonal wurtzite ZnO, respectively [32,33]. The most intense peak at ~42.3° is indexed to the (101) plane, suggesting a preferential growth orientation along this direction [32]. All reflections match the standard pattern for wurtzite ZnO (XRD JCPDS No. 01-080-0075), and no impurity peaks are detected, confirming high phase purity. The average crystallite size was estimated using Pawley refinement with a Double-Voigt approach. The volume-weighted crystallite size (LVol-IB) was determined to be approximately 26.2 ± 0.6 nm, while the LVol-FWHM value was about 34.7 ± 1.0 nm, confirming the nanocrystalline nature.

The FTIR spectrum (Figure 1b) shows characteristic Zn–O vibrational bands at 417 and 572 cm^−1^ [34]. A broad band around 3433 cm^−1^ is attributed to O–H stretching from adsorbed moisture. The chemical state and surface composition were analyzed by XPS. The high-resolution Zn 2p spectrum (Figure 1c) displays two peaks at 1022.2 eV (Zn 2p_3/2_) and 1045.2 eV (Zn 2p_1/2_), with a spin–orbit splitting of 23 eV, characteristic of Zn^2+^ in the ZnO lattice [35,36]. The O 1s spectrum (Figure 1d) is dominated by a peak at 530.8 eV, assigned to lattice oxygen (O^2−^) in Zn–O bonds. The absence of signals from metallic Zn or other oxides indicates high chemical purity.

The BET specific surface area was determined to be 21.29 m^2^ g^−1^, which is typical for nanocrystalline ZnO synthesized by chemical methods [29]. The SEM micrograph (Figure 1f) reveals plate-shaped two-dimensional nanoparticles (nanoplates) with an average size of 208 × 97 nm (determined from 50 nano-objects), forming irregular aggregates (a common behavior due to the high surface energy of ZnO nanostructures) [29,37]. The aggregates consist of densely packed primary nanoplates. This moderate aggregation correlates with the measured surface area, indicating a relatively accessible surface.

### 2.2. Dense Membrane Investigation

#### 2.2.1. Structural and Physicochemical Characterization of Membranes

**Figure 2:**
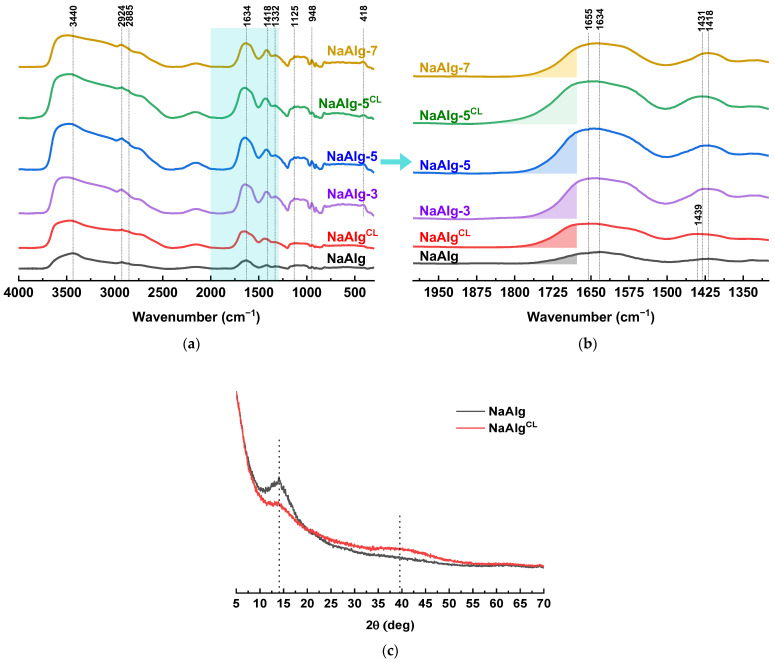
(**a**) FTIR spectra of the dense membranes, both cross-linked and uncross-linked, in the range 4000–400 cm^−1^; (**b**) FTIR spectra in the range 2000–400 cm^−1^; (**c**) wide-angle XRD patterns of the NaAlg and NaAlg^CL^ membranes.

The structure of the dense membranes, both cross-linked (NaAlg^CL^ and NaAlg-5^CL^) and uncross-linked (NaAlg, NaAlg-3, NaAlg-5, and NaAlg-7), was studied using FTIR and ^13^C NMR spectroscopy. The FTIR spectrum of NaAlg (Figure 2a) displays characteristic bands at 3440 cm^−1^ (O–H stretching), 2924 and 2855 cm^−1^ (C–H stretching), 1634 and 1418 cm^−1^ (asymmetric and symmetric stretching of carboxylate groups, respectively), 1332 and 948 cm^−1^ (C–O), and 1125 cm^−1^ (C–C) [38]. Incorporation of ZnO nanoplates introduced a new band at ~418 cm^−1^, corresponding to Zn–O bond stretching, which aligns with the band position observed in the FTIR spectrum of pristine ZnO (Figure 1b). Furthermore, in the composite membranes, the carboxylate asymmetric stretching band exhibits a shoulder on the high-frequency side, as well as a minor shift of the peak maximum from 1634 cm^−1^ for NaAlg to 1639, 1644, and 1638 cm^−1^ for NaAlg-3, NaAlg-5, and NaAlg-7, respectively (Figure 2b). This shift suggests an interaction between the zinc metal centers and the carboxylate groups of the alginate. For NaAlg-7, this effect is less pronounced, which is likely due to particle agglomeration and poor dispersion within the polymer matrix (as shown by the SEM micrographs below), limiting the number of possible interactions.

**Figure 3:**
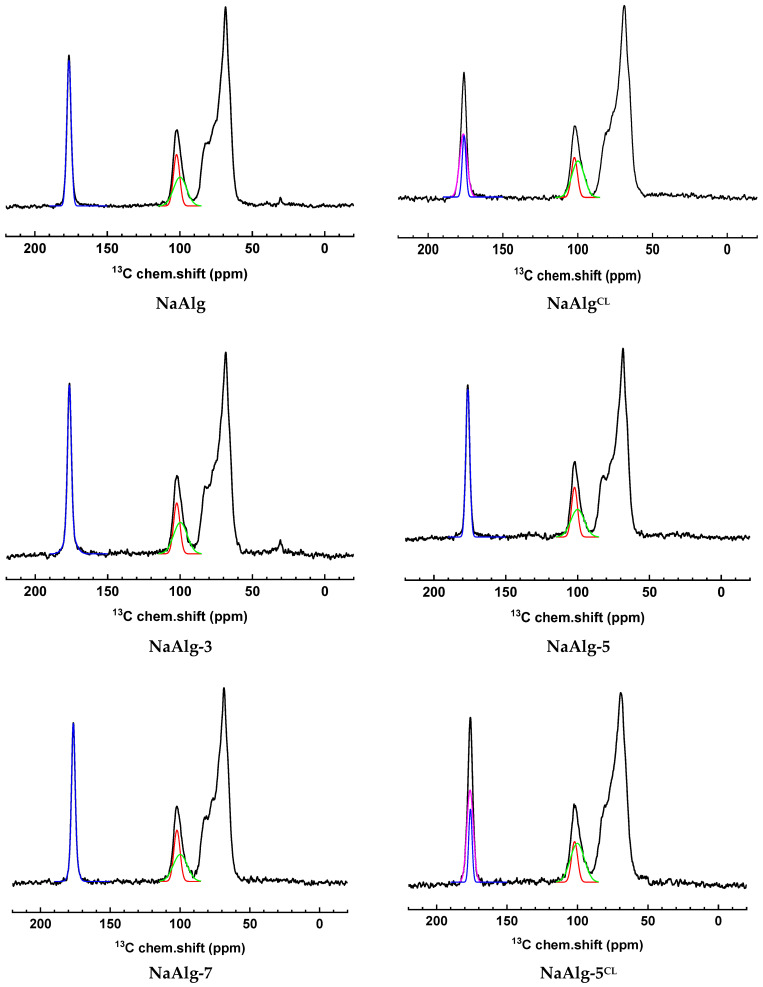
^13^C NMR spectra of the dense membranes, both cross-linked and uncross-linked.

Ion-exchange cross-linking with CaCl_2_, which forms calcium alginate (CaAlg) according to the “egg-box” model, was confirmed by significant shifts of the carboxylate bands [39,40]. The characteristic asymmetric/symmetric stretching doublet shifted from 1634/1418 cm^−1^ in pristine NaAlg to 1655/1439 cm^−1^ for NaAlg^CL^ and to 1655/1431 cm^−1^ for NaAlg-5^CL^. These spectroscopic findings are supported by the corresponding XRD and NMR data (Figure 2c and Figure 3).

The XRD pattern of NaAlg reveals a predominantly amorphous material featuring a broad halo at ~39.5° and a weak peak at 14.0°, the latter associated with the (110) plane of polyguluronate segments [41]. Ion-exchange cross-linking resulted in a further decrease in crystallinity. This less-ordered structure of cross-linked polymer matrix, as confirmed by the following NMR results, enhances water molecule diffusion, which contributes to the increased permeation flux of cross-linked membranes compared to their uncross-linked counterparts.

The ^13^C NMR spectra are presented in Figure 3. The spectra contain all expected resonances characteristic of NaAlg [9]. The broadened line near 100 ppm can be deconvoluted into two components corresponding to the amorphous (green color in Figure 3) and crystalline (red color in Figure 3) packing of the polymer. The degree of crystallinity, estimated from the ratio of their areas, was 40% for NaAlg and decreased to 35% for NaAlg^CL^. This reduction upon cross-linking aligns with the XRD results (Figure 2c). Furthermore, for cross-linked membranes, the line corresponding to carboxyl carbons broadened, which can be modeled by adding a second spectral component (magenta color in Figure 3) likely arising from alginate units coordinated to calcium ions. The cross-linking degree, estimated from the area ratio of these two components, was comparable: 67% for NaAlg^CL^ and 70% for NaAlg-5^CL^.

Notably, the introduction of ZnO into the NaAlg matrix increased the crystallinity to 43%, 45%, and 46% for NaAlg-3, NaAlg-5, and NaAlg-7, respectively, likely due to polymer–modifier interactions, as confirmed by FTIR (Figure 2b) and DFT calculations (Section 2.2.2). A similar effect of a nanofiller enhancing polymer matrix crystallinity has been previously observed for a NaAlg/fullerenol system [9]. Furthermore, nanoparticles have been reported to act as nucleating agents, promoting polymer crystallization and increasing the overall crystallinity, as shown by A. Abdali et al. [21]. However, for the cross-linked modified membrane (NaAlg-5^CL^), the crystallinity decreased to 32%. This can be attributed to the hydrophilic nature of ZnO, as indicated by contact angle measurements (Table 1) and supported by literature [28], combined with an increased surface roughness (confirmed by AFM below). The latter contributes to a greater number of sorption sites. These factors collectively enhance the efficiency of ion exchange during the cross-linking process, ultimately resulting in a higher cross-linking degree.

**Figure 4:**
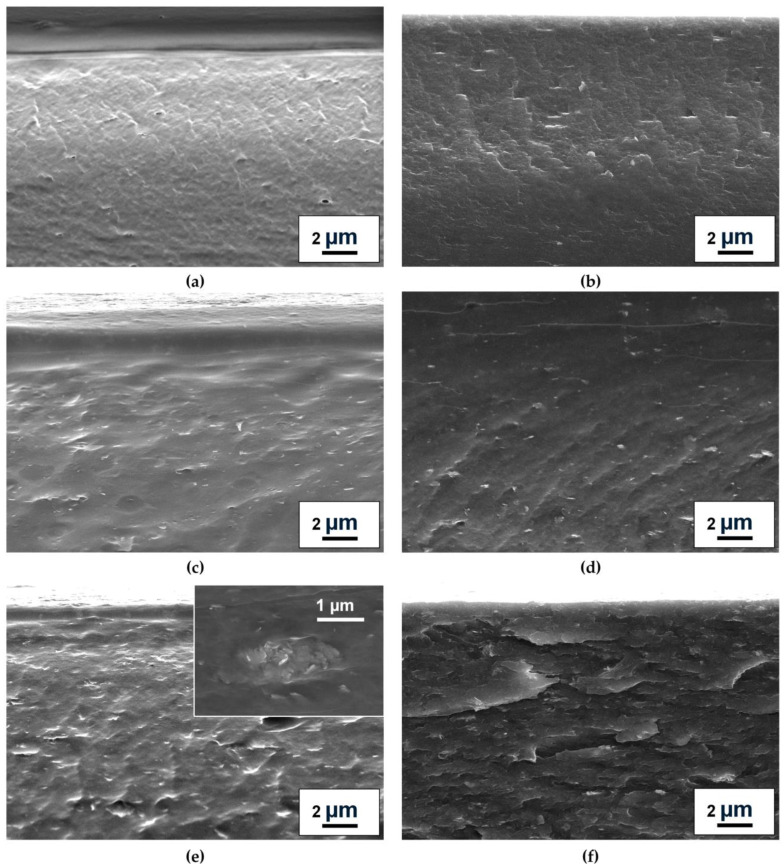
Cross-sectional SEM micrographs of the dense membranes: (**a**) NaAlg, (**b**) NaAlg^CL^, (**c**) NaAlg-3, (**d**) NaAlg-5, (**e**) NaAlg-7, and (**f**) NaAlg-5^CL^.

**Figure 5:**
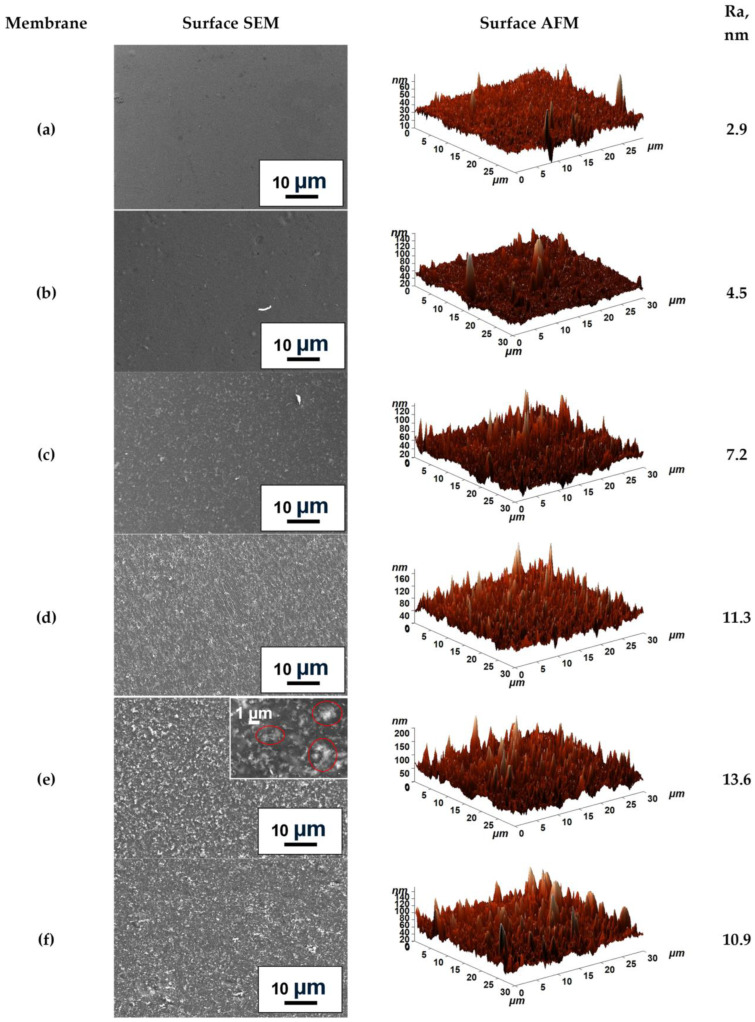
Surface SEM micrographs, surface AFM images, and average roughness of the dense membranes: (**a**) NaAlg, (**b**) NaAlg^CL^, (**c**) NaAlg-3, (**d**) NaAlg-5, (**e**) NaAlg-7, and (**f**) NaAlg-5^CL^.

Changes in membrane morphology and surface topography upon ZnO addition and cross-linking were studied by SEM and AFM. Cross-sectional and surface SEM micrographs, along with AFM images (30 × 30 µm scan size) and calculated average roughness (Ra) values, are presented in Figure 4 and Figure 5.

The pristine NaAlg membrane exhibited a smooth, uniform cross-section and surface topography. For the composites, good dispersion of the modifier within the polymer matrix and significant morphological changes were observed, indicating polymer–modifier interfacial interactions consistent with the FTIR spectral shift (Figure 2b) and the additional NMR spectral component (Figure 3). Increasing the modifier loading from 3 to 5 wt.% led to a corresponding increase in visible nano-objects in SEM micrographs and surface roughness (Ra from 7.2 to 11.3 nm). However, at 7 wt.% (NaAlg-7), moderate agglomeration of ZnO particles (up to 2 µm) is observed in cross-sectional SEM, which likely would contribute to the reduced permeation flux of NaAlg-7 (Section 2.2.3) [20]. Cross-linking with CaCl_2_ induced a flake-like morphology in the membranes, which aligns with previous findings [7,42,43]. It is also worth noting that the introduction of the modifier led to an increase in membrane thickness, as illustrated by the cross-sectional SEM micrographs presented in Figure S1 of the Supporting Information. Specifically, for the NaAlg-3, NaAlg-5, and NaAlg-5^CL^ samples, the average thickness was 34 ± 5 µm, compared to 30 ± 5 µm for NaAlg and NaAlg^CL^. Meanwhile, the NaAlg-7 membrane exhibited the greatest thickness at 41 ± 3 µm, which is likely attributable to the formation of agglomerates and contributed to the reduction in permeation flux.

Unmodified membranes (NaAlg, NaAlg^CL^) showed uniform surface topography with low Ra values of 2.9 and 4.5 nm, respectively. Incorporation of ZnO created a more developed surface, with Ra increasing proportionally with filler loading [42,44]. NaAlg-7 exhibited surface agglomerates (red circles in Figure 5e), consistent with cross-sectional micrographs and the highest Ra value (13.6 nm). Notably, NaAlg-5^CL^ and NaAlg-5 showed similar Ra values (10.9 vs. 11.3 nm, within a ~5% relative error), suggesting that the influence of ZnO nanoplates on surface topography dominates over the structural reorganization induced by cross-linking.

The liquid uptake and water contact angles for the cross-linked dense membranes are summarized in Table 1. As a result of CaCl_2_ cross-linking, all membranes remained stable in water for 28 days. Additionally, LU curves as a function of water content across the entire concentration range of the H_2_O/IPA mixture were obtained (Figure S3 of the Supporting Information). The incorporation of ZnO led to a slight increase in LU values compared to the unmodified NaAlg^CL^ membrane. This enhancement is attributed to the reduced crystallinity of the polymer phase (as determined by NMR, Figure 3), an effect likely caused by the hydrophilic nature of ZnO. The modifier increases the number of sorption sites and promotes more efficient ion exchange during the cross-linking process. It should also be noted that the low LU values for IPA are indicative of the membrane’s high selectivity for water. This preference likely contributes to the high water content observed in the permeate.

Pristine, uncross-linked membranes were unsuitable for contact angle measurements, as they underwent immediate dissolution upon droplet contact. The data indicate a pronounced shift towards greater hydrophilicity for the NaAlg-5^CL^ membrane compared to NaAlg^CL^. This shift is directly attributed to the presence of hydrophilic ZnO nanoplates on the surface [28]. The increased water affinity correlates with the higher liquid uptake and the consequent rise in permeation flux (Section 2.2.3). This trend is further supported by DFT calculations, which revealed favorable non-covalent interactions between ZnO and water molecule.

**Figure 6:**
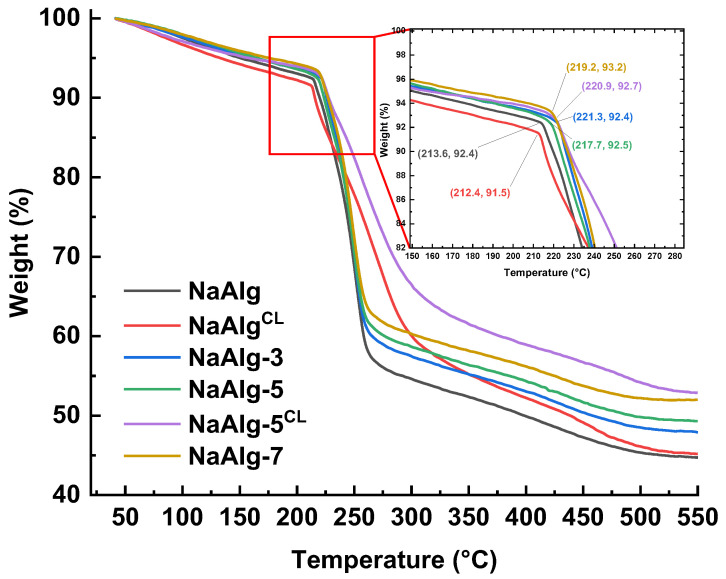
Thermogravimetric curves for the uncross-linked and cross-linked dense membranes.

The thermal stability of the dense membranes was assessed by TGA. The TGA curves (Figure 6) show three main stages of mass loss in the temperature range of 30 to 550 °C.

The first stage (up to ~220 °C) corresponds to the removal of residual moisture from the hydrophilic membrane material [45]. Compared to the unmodified NaAlg membrane, the composite membranes (NaAlg-3, NaAlg-5, NaAlg-7) showed a higher termination temperature for this step. This can be attributed to interactions between NaAlg and ZnO nanoplates, as confirmed by FTIR spectroscopy (Figure 2b), indicating enhanced thermal stability. Conversely, the cross-linked NaAlg^CL^ membrane exhibited the greatest mass loss in this region, likely due to the swelling of the polymer matrix with water during the immersion in the CaCl_2_ solution.

The second step (220–300 °C) is assigned to the decomposition of functional groups and dehydration of polysaccharide bonds [9]. Notably, the profiles of the cross-linked membranes (NaAlg^CL^ and NaAlg-5^CL^) differ significantly from those of the uncross-linked ones, displaying lower mass loss up to 300 °C. This is associated with the formation of the stable “egg-box” structure, as confirmed by ^13^C NMR (Figure 3).

The final step involves the complete degradation of the polymer backbone, forming carbonaceous residues and carbonates (Na_2_CO_3_/CaCO_3_) [45]. The difference in total weight loss for NaAlg-3, NaAlg-5, and NaAlg-7 compared to pristine NaAlg was 2.8, 4.7, and 7.3 wt.%, respectively. These values are in good agreement with the nominal ZnO loadings and are a direct consequence of the high thermal stability of the ZnO filler. It is noteworthy that the NaAlg-5^CL^ membrane demonstrates the lowest overall mass loss. This is likely a synergistic result of three factors: (i) the 5 wt.% loading of thermally stable ZnO, (ii) ionic cross-linking with CaCl_2_, and (iii) non-covalent interactions between ZnO and the polymer matrix, which together alter the composite’s structure and morphology.

#### 2.2.2. DFT Investigation of Noncovalent Interactions

Water, isopropanol (IPA), a ZnO molecule, and monomeric units of NaAlg, namely the sodium salts of mannuronic acid (^M^NaAlg) and guluronic acid (^G^NaAlg), were used as model species. Based on the optimized geometries of these individual molecules, a set of 1:1 associates was constructed to evaluate the resulting non-covalent interactions. The Cartesian coordinates of the optimized individual molecules and associates are provided in Table S1 of the Supporting Information. The changes in thermodynamic potentials were calculated as the difference between the energy of the associate and the sum of the energies of its initial components. The calculated changes in enthalpy and Gibbs free energy are presented in Table S2 of the Supporting Information. Table 2 summarizes the change in the isothermal-isobaric potential (ΔG) for the most stable associates.

The obtained values indicate a high thermodynamic favourability for the interaction of both the polymer matrix and the modifier with the feed components. This is primarily driven by the formation of coordination clusters involving sodium ions [46]. Similar behavior has been reported in earlier studies for interactions with benzene-1,4-dicarboxylic acid and benzene-1,3,5-tricarboxylic acid [47,48]. Compared to previous lower-level computational studies, the application of a higher-level basis set combined with the D3(BJ) empirical dispersion correction resulted in a more favorable binding energy for the IPA complex relative to the water complex with the alginate monomers. For IPA interacting with ^M^NaAlg and ^G^NaAlg, this is linked to the formation of three intermolecular interactions per associate. In the case of ZnO, the difference can be explained by the positive inductive effect of the isopropyl group, which donates electron density to the oxygen atom, leading to the formation of a stronger Zn···O(HO–C_3_H_7_) bond, as confirmed by bond order analyses (Table S3 of the Supporting Information) and a shorter interatomic distance. The interaction of only the hydroxy groups of ^M^NaAlg with water is more favorable than with IPA (7.1 and 9.5 kJ mol^−1^, respectively, Table S3 of the Supporting Information). This fact, combined with water’s smaller molecular size, lower steric hindrance, and its ability to form an extensive hydrogen-bonding network, underlies the observed high selectivity of NaAlg membranes toward water.

The interaction of ZnO with the feed components is characterized by significantly higher (more negative) ΔG values. This aligns with the experimentally observed increase in hydrophilicity (lower water contact angle) and liquid uptake (Table 1), which facilitates the sorption and diffusion of both water and IPA, thereby contributing to the increased permeation flux of modified membranes.

**Figure 7:**
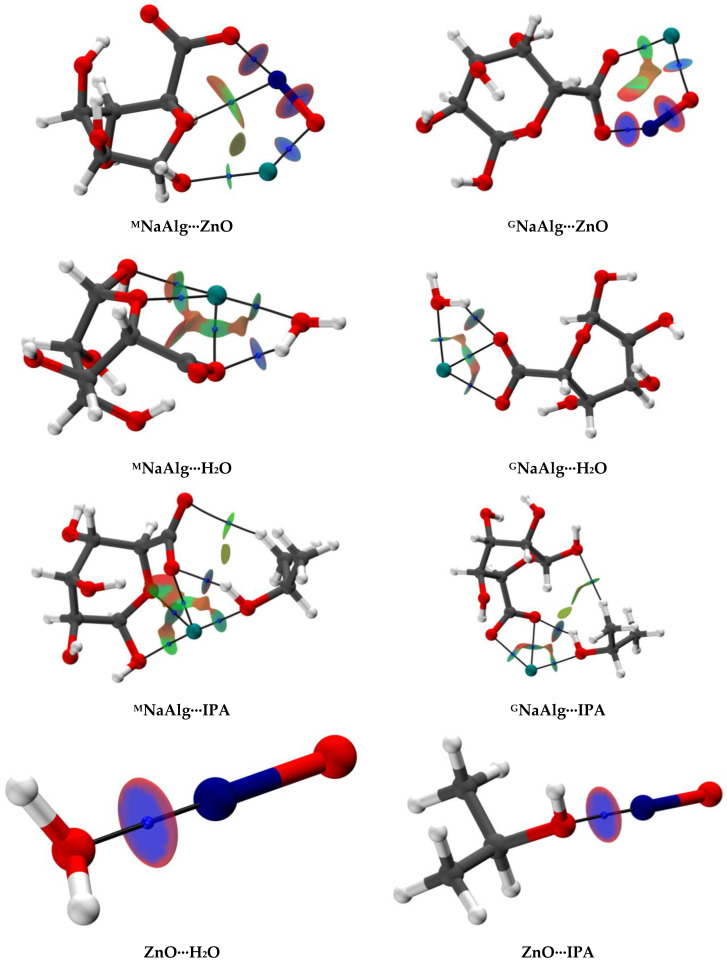
Optimized structures of the model associates, with NCI plots, bond critical points and bond paths.

To confirm the formation of non-covalent interaction, determine their attractive type, and assess the strength, a topological analysis of the electron density was performed using the Quantum Theory of Atoms in Molecules (QTAIM), along with the generation of non-covalent interaction plots and bond order analyses [49,50,51]. Figure 7 presents the most stable associates with Bond Critical Points (BCPs, blue spheres) and bond paths (black lines), which visually confirm the formation of non-covalent interactions.

The topological analysis indicates that the carboxylate group can interact with either the Na^+^ ion or the Zn atom, suggesting these centers compete for the same binding site. This is consistent with the frequent use of Zn^2+^ ions as cross-linking agents [52] and is supported by the shift of the carboxylate asymmetric stretching band in the FTIR spectrum of NaAlg-5 and the increase in crystallinity shown by NMR (Figure 2b and Figure 3). This interaction is likely responsible for the change in cross-sectional morphology observed by SEM (Figure 4). Based on Wiberg Bond Indices (WBI) (Table S3 of the Supporting Information) and NCI plots (Figure 7), all considered O–H interactions can be characterized as hydrogen non-covalent interactions. For the Na^+^ cation in NaAlg, WBIs greater than 0.3 were observed, and the ratio of the interatomic distance to the sum of the van der Waals radii was less than 60%, which indicates an interaction of an intermediate nature between a non-covalent bond and a dative bond [53,54,55]. Finally, when the ZnO molecule interacts with the monomeric NaAlg units, it integrates into the sodium coordination sphere, forming a strong Zn–O(COO^−^) bond. According to WBI values of 0.845 (^G^NaAlg) and 0.941 (^M^NaAlg), this interaction can be classified as a coordination bond. This leads to a reorganization of the monomer unit structure and a significant change in Gibbs free energy (Table 2). The higher WBI value for ^M^NaAlg is associated with an additional interaction between the Zn and an oxygen atom of the pyranose ring.

#### 2.2.3. Transport Properties of Membranes

**Figure 8:**
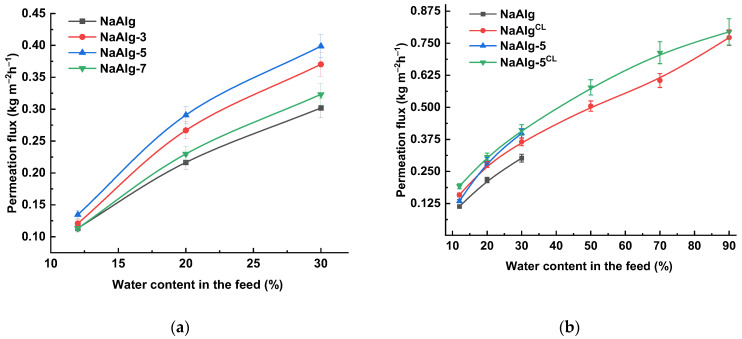
Permeation flux as a function of water content in the feed for (**a**) membranes with different ZnO loadings and (**b**) cross-linked and uncross-linked membranes from NaAlg and NaAlg-ZnO (5%) composite. The water content in the permeate was 99.99 wt.%.

The transport properties of the dense uncross-linked membranes were evaluated in the pervaporation separation of IPA/water mixtures with water contents ranging from 12 to 30 wt.%. The results are presented in Figure 8a. All synthesized membranes exhibited high selectivity towards water (99.99 wt.% water in the permeate).

The unmodified NaAlg membrane demonstrated a permeation flux ranging from 0.113 to 0.302 kg m^−2^h^−1^ when separating feed with water concentrations from 12 to 30 wt.%, respectively. Attempts to use feeds with higher water content led to excessive, uncontrolled swelling of the NaAlg matrix. Incorporating from 3 to 7 wt.% of ZnO into the polymer matrix resulted in an increase in permeation flux compared to pristine NaAlg. According to the classic solution-diffusion model, separation performance is governed by both the sorption and diffusion of individual components [56]. The introduction of ZnO led to an increase in surface roughness (confirmed by AFM images, Figure 5c–f) and surface hydrophilicity (supported by DFT-based bond order analysis of hydrogen bonds). These modifications increase the accessible surface area and improve the interfacial contact with the feed components. The observed changes can be explained by the presence of hydrophilic ZnO particles on the membrane surface, as evidenced by surface SEM micrographs in Figure 5e. The combined factors of greater roughness and hydrophilicity enhance the sorption capacity for the feed components, contributing to the observed increase in permeation flux [57]. Concurrently, cross-sectional SEM micrographs (Figure 4) revealed significant morphological alterations induced by interactions between the carboxylate groups of NaAlg and ZnO nanoplates, as confirmed by FTIR spectroscopy (Figure 2b) and DFT calculations. These interactions also resulted in an increase in the crystallinity of the NaAlg-5 phase, as determined by NMR spectroscopy (Figure 3). While this enhanced crystallinity helps maintain high selectivity, it may also contribute to a reduction in permeation flux [21]. However, literature reports indicate that nanoparticles can facilitate penetrant diffusion by creating additional transport pathways at the polymer–filler interface [20]. These findings are in agreement with the sequential permeation flux increase observed for the 3 and 5 wt.% ZnO loadings. Finally, for the NaAlg-7 sample, a decrease in permeation flux was observed while selectivity was maintained. This may be attributed both to an increase in membrane thickness due to the formation of agglomerates (as shown by cross-sectional SEM micrographs in Figure S1 of the Supporting Information) and to constraints on molecular transport resulting from blockage by the nonporous solid ZnO nanoplates. Specifically, this effect has been noted in the work of Lu and Suen [13], in which it was reported that at high filler loadings, the negative contribution may outweigh the factors that enhance permeation flux. Based on the obtained transport characteristics, the NaAlg-5 membrane was identified as optimal, and this composition was selected for developing the cross-linked NaAlg-5^CL^ membrane.

Ionic cross-linking with CaCl_2_ provided water stability to the membranes, enabling the separation of feeds with high water content (up to 90 wt.%) (Figure 8b). Furthermore, the cross-linked membranes showed an increased permeation flux compared to uncross-linked NaAlg. This enhancement is attributed to the reduction in polymer crystallinity, as confirmed by NMR (Figure 3) and XRD (Figure 2c), and is consistent with the results from swelling experiments (Table 1). It is noteworthy that for the 12 wt.% water feed, the permeation flux of NaAlg-5 (0.135 kg m^−2^h^−1^) was lower than that of NaAlg^CL^ (0.159 kg m^−2^h^−1^). This difference can be explained by the lower crystallinity of NaAlg^CL^ (NMR data) and the extremely limited swelling of the polymer matrix in IPA (liquid uptake studies in Table 1). However, at 90 wt.% water in the feed, the difference in permeation flux for the cross-linked membranes becomes smaller, which is likely due to significant swelling of the polymer matrix (as shown by LU experiments, Figure S3 of the Supporting Information). The best overall performance was achieved by the NaAlg-5^CL^ membrane, which exhibited permeation fluxes from 0.192 to 0.794 kg m^−2^h^−1^ while maintaining high water selectivity (99.99 wt.%). This membrane also featured the lowest crystallinity and a more developed, hydrophilic surface compared to the NaAlg^CL^ membrane.

### 2.3. Supported Membrane Investigation

**Figure 9:**
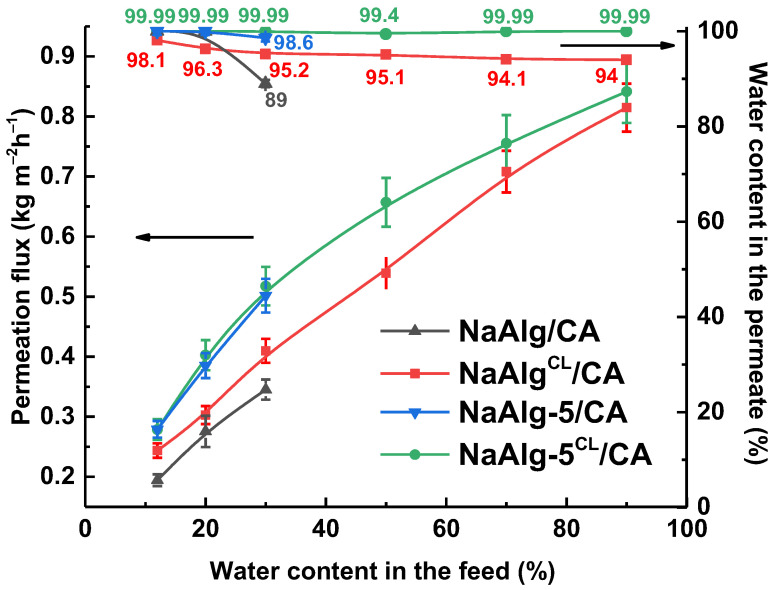
Permeation flux and water content in the permeate as a function of water concentration in the feed for the supported membranes.

A widely used strategy to enhance separation efficiency in industrial applications involves fabricating supported membranes. This architecture consists of a thin, selective layer deposited on a porous substrate: the selective layer determines separation performance, while the substrate offers essential mechanical integrity. A central research challenge is optimizing this combination to maximize synergy between the layers without introducing additional resistance to mass transport [46]. In this study, supported membranes were fabricated by casting a NaAlg-based selective layer onto a porous CA substrate. The performance of the developed cross-linked supported membranes (NaAlg^CL^/CA and NaAlg-5^CL^/CA) was evaluated in the pervaporation separation of water/IPA mixtures with water concentrations from 12 to 90 wt.% (Figure 9). Additionally, for comparison, the corresponding uncross-linked NaAlg/CA and NaAlg-5/CA membranes were prepared and tested.

The modified NaAlg-5^CL^/CA membrane showed superior performance, achieving a permeation flux between 0.279 and 0.841 kg m^−2^h^−1^, which was higher than for the unmodified NaAlg^CL^/CA membrane. The incorporation of 5 wt.% ZnO improved the permeation flux by 33 and 26% for feeds containing 20 and 30 wt.% water, respectively. Furthermore, the NaAlg-5^CL^/CA membrane maintained a high water content in the permeate (>99 wt.%) across the whole concentration range, which is likely attributable to the increase in crystallinity upon ZnO incorporation (as confirmed by NMR, Figure 3). In contrast, the water content in the permeate for the NaAlg^CL^/CA membrane gradually decreased to 94 wt.% at higher feed water concentrations. For the uncross-linked NaAlg/CA and NaAlg-5/CA membranes, similar transport characteristics trend was observed. However, after exposure to 30 wt.% water in the feed, a deterioration in transport properties was noted—though these results were not reproducible—primarily due to excessive swelling and water solubility of the NaAlg-based selective layer. These changes were less pronounced in the membranes containing the modifier.

Despite a significant reduction in the active layer thickness from approximately 30 µm (dense membranes) to about 250–280 nm (supported membranes), the gain in permeation flux was limited. The permeation flux increased by 2 or more times for the NaAlg-5^CL^/CA membrane compared to the dense NaAlg membrane when dehydrating IPA to 30 wt.% water. This deviation from the theoretical inverse proportionality to thickness can be ascribed to the influence of the porous substrate structure. Potential contributing factors include partial pore blockage by the polymer solution during coating, which could alter the hydraulic resistance of the substrate, and the possible formation of small defects within the ultrathin selective layer during fabrication or operation [46]. These factors may modify the effective driving force or create non-selective pathways, offsetting the expected flux or separation enhancement from thickness reduction.

**Figure 10:**
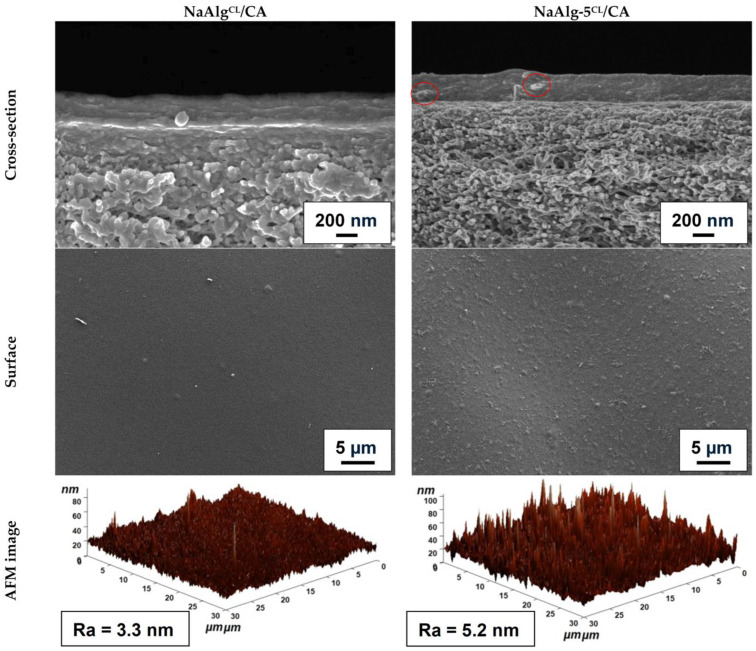
Surface and cross-section SEM micrographs, surface AFM images, and average roughness of the supported membranes.

The structure and surface topography of the supported NaAlg^CL^/CA and NaAlg-5^CL^/CA membranes were characterized by SEM and AFM. Surface and cross-sectional SEM micrographs, AFM images, and average surface roughness are presented in Figure 10.

The CA support exhibits a spongy-like morphology with large vacuoles (Figure S2 of the Supporting Information), which is consistent with the morphology previously described [58]. Analysis of the cross-sectional SEM micrographs provided the thickness of the selective layer, which was approximately ~280 nm for NaAlg^CL^/CA and ~250 nm for NaAlg-5^CL^/CA. In contrast to the significant morphological changes observed in the dense membranes (Figure 4), the cross-sectional micrographs of the supported membranes appeared similar, indicating good adhesion between the selective layer and the porous CA support. However, individual ZnO nano-objects were visible on both the cross-section and the surface of the NaAlg-5^CL^/CA membrane (red circles in Figure 10), confirming the uniform distribution of the modifier.

The AFM results supported the SEM observations. The incorporation of 5 wt.% ZnO increased the Ra. This increase enhances the effective surface area and the number of hydrophilic sorption sites (as confirmed by the decrease in water contact angle from 65° for NaAlg^CL^/CA to 45° for NaAlg-5^CL^/CA), contributing to the higher permeation flux [57]. Although the supported membranes exhibited a similar surface modification trend to the dense membranes, their absolute Ra values were lower. This reduction can be attributed to the influence of the underlying CA support’s smoother surface topography (Ra = 0.8 nm [58]) and the potential partial clogging of its pores by the selective layer [46]. Consequently, the selective layer likely replicates the substrate’s morphology while also infiltrating its pores, which moderates the expected rise in permeation flux.

### 2.4. Comparison of NaAlg-Based Membrane Performance

Table 3 compares the transport properties of the developed NaAlg-based membranes with those reported in the literature for the separation of a water/IPA azeotropic mixture.

As shown, the fabricated membranes demonstrate promising performance due to their high permeation flux and/or selectivity, which suggests their competitiveness against a commercial analogue. For the supported membranes, the developed NaAlg-5^CL^/CA membrane exhibits improved permeability compared to the commercial PERVAP™ 1201 membrane.

## 3. Materials and Methods

### 3.1. Materials

Sodium alginate (NaAlg), supplied by Jiangsu Benefit Ocean Technology Co., Ltd. (Lianyungang, China), was used as the membrane matrix. Synthesized zinc oxide (ZnO) nanoplates served as the membrane modifier. Zinc acetate dihydrate (Zn(CH_3_COO)_2_·2H_2_O, purity ≥98%) and potassium hydroxide (KOH, purity ≥99%, pellets), both supplied by “Vekton” (St. Petersburg, Russia), were utilized as precursors for the nanoparticle synthesis. Cellulose diacetate (CA, Mn = 40,000 g mol^−1^) from CJSC “Vladipor” (Vladimir, Russia) was employed as the porous substrate matrix for the development of supported (composite) membranes. N,N-Dimethylacetamide (DMAc, puriss.) and isopropyl alcohol (IPA, puriss.), both from “Vecton” (Saint Petersburg, Russia), were used as the solvent for the CA solution and as a component of the feed, respectively. Calcium chloride dihydrate (CaCl_2_·2H_2_O, puriss.) was purchased from “NevaReaktiv” (St. Petersburg, Russia). All chemicals were used as received.

### 3.2. ZnO Synthesis

ZnO nanoplates were synthesized via a method described in the work by R. Khokhra et al. [37]. An aqueous solution of Zn(CH_3_COO)_2_·2H_2_O (0.4 M, 100 mL) was stirred magnetically. Subsequently, a KOH solution (2 M, 100 mL) was added dropwise over approximately 10 min, inducing the immediate formation of a white precipitate. The resultant suspension was aged for 2 h at room temperature to allow for complete particle growth and crystallization. The precipitate was isolated by vacuum filtration, washed repeatedly with deionized water until the filtrate reached a neutral pH, and then dried in an oven at 60 °C for 12 h.

### 3.3. ZnO Characterization

The phase composition and crystal structure of the synthesized ZnO nanoplates were characterized by XRD, FTIR spectroscopy, and X-ray photoelectron spectroscopy (XPS). XRD patterns were collected in the 2θ range of 5.0–105.0° with a step size of 0.0506° (30 kV, 10 mA) and analyzed using Pawley refinement combined with a Double-Voigt approach. Phase identification was performed using the ICDD PDF-2 database (Release 2016). FTIR spectrum was recorded between 450 and 4000 cm^−1^ at 25 °C. XPS analysis was conducted on an Escalab 250Xi spectrometer (Thermo Fisher Scientific, Waltham, MA, USA) using monochromatic AlKα radiation (1486.6 eV). Measurements were performed with a constant pass energy of 20 eV, a spot size of 650 μm, and an energy resolution of ~0.8 eV. A charge compensation system was employed to neutralize surface charging. All spectra were acquired at room temperature under ultrahigh vacuum (10^−9^ mbar) and calibrated with a binding energy accuracy of ±0.3 eV.

The specific surface area was determined from low-temperature nitrogen adsorption isotherm measured on an ASAP 2020 analyzer (Micromeritics Instrument Corp., Norcross, GA, USA). Prior to measurement, the sample was degassed under vacuum. The surface area was calculated using the Brunauer–Emmett–Teller (BET) method within the standard relative pressure range. Morphological evaluation of ZnO nano-objects was performed by scanning electron microscopy (SEM) using the Zeiss AURIGA Laser (Carl Zeiss SMT, Oberhochen, Germany).

### 3.4. Membrane Preparation

A NaAlg solution was prepared by dissolving a measured quantity of NaAlg (1 g) in 99 g of deionized water. The mixture was stirred for 4 h at 45 °C until a homogeneous solution was obtained. To prepare the composite, NaAlg was ground in an agate mortar with a predetermined amount of ZnO (3–7 wt.% relative to the polymer mass) for 40 min, followed by dissolution in water using the procedure described above. Immediately after preparation, it was then subjected to ultrasonication at room temperature (35 kHz, 15 min) and cast onto Petri dishes. NaAlg-based membranes were fabricated via the solvent evaporation in a convection oven at 40 °C for 24 h. The thickness of the resulting dense membranes was 30 ± 5 μm, as measured with a micrometer (Schut Geometrical Metrology, Trossingen, Germany).

Porous substrates fabricated from 15 wt.% CA solution via the non-solvent induced phase separation (NIPS) technique, as described and characterized in [58], were used as supports for the membranes with a dense selective layer (composite or supported). These porous CA substrates were coated with either pristine NaAlg solutions or NaAlg-ZnO composite and subsequently air-dried for 24 h to obtain the supported membranes.

Cross-linking was performed by immersing the prepared dense and supported membranes in a 1.25 wt.% aqueous CaCl_2_ solution for 5 min at room temperature. The resulting cross-linked membranes were rinsed with distilled water and dried at 25 °C. The denotations of the prepared NaAlg-based membranes and their compositions are summarized in Table 4.

### 3.5. Membrane Characterization

Membrane transport properties were evaluated in a vacuum pervaporation conditions at 25 °C. A membrane sample with an effective area of 9.6 cm^2^ was placed in a laboratory-scale dead-end pervaporation cell. The experimental setup and a detailed description of the methodology are provided in [47]. The permeate vapor was condensed in a trap cooled with liquid nitrogen, and its composition was analyzed by gas chromatography using a Chromatec Crystal 5000.2 chromatograph (Chromatec, Yoshkar-Ola, Russia) equipped with a Hayesep R column (2 m × 3 mm) and a thermal conductivity detector.

The membrane performance was presented in terms of two key transport parameters: the permeation flux (J, Equation (1)) and the separation factor (β, Equation (2)).

(1) $$J=\frac{\Delta m}{S*\Delta t}\;\;,$$

where Δm (kg) is the mass of the collected permeate, S (m^2^) is the effective membrane area, and Δt (h) is the permeate collection time.

(2) $$\beta_{H_{2}O/IPA}=\frac{Y_{H_{2}O}/Y_{IPA}}{X_{H_{2}O}/X_{IPA}\;\;},$$

where $Y_{i}$ and $X_{i}$ represent the weight fractions of component i (water or IPA) in the permeate and feed solutions, respectively.

Each experiment was conducted in at least five replicates. The obtained values were averaged, and the confidence intervals for the permeation flux and separation factor were calculated based on the normalized standard distribution (*p* = 0.95).

The fabricated membranes were subjected to a suite of analytical techniques to determine their structural and physicochemical properties. Chemical structure and functional groups were characterized using Fourier-transform infrared (FTIR) spectroscopy (IRAffinity-1S, Shimadzu, Kyoto, Japan) across 400–4000 cm^−1^ and solid-state ^13^C nuclear magnetic resonance (NMR) spectroscopy. NMR spectra were recorded on a Bruker Avance III 400 WB spectrometer (Billericay, MA, USA, 9.4 T) equipped with a 4 mm CP/MAS probe, operating at a magic angle spinning rate of 10 kHz and a ^13^C resonance frequency of 100.64 MHz, with tetramethylsilane as an external reference.

Crystallinity was assessed by X-ray diffraction (XRD) on a Bruker D8 DISCOVER diffractometer (Bremen, Germany) using CuKα radiation (λ = 1.5406 Å), scanning 2θ from 5° to 70° with a step of 0.0502°. Morphological evaluation of surfaces and cryo-fractured cross-sections was performed by scanning electron microscopy (SEM, Zeiss AURIGA Laser, Carl Zeiss SMT, Oberhochen, Germany) using In-lens and SE2 detectors. Surface topography and roughness parameters were obtained via atomic force microscopy (AFM, NT-MDT NTegra Maximus, NT-MDT Spectrum Instruments, Moscow, Russia) in tapping mode with silicon probes (spring constant 15 N m^−1^).

Liquid uptake behavior (sorption, swelling) of cross-linked dense membranes was determined gravimetrically. Samples were immersed in H_2_O, IPA, and H_2_O/IPA mixtures at 25 °C until swelling equilibrium was reached. The liquid uptake (LU) was calculated according to Equation (3):

(3) $$LU=\frac{m_{s}-m_{d}}{m_{d}},$$

where $m_{s}$ (g) is the mass of the swollen membrane, and $m_{d}$ (g) is the mass of the same sample after drying in a vacuum oven at 50 °C for 24 h.

Surface hydrophilicity was quantified by water contact angle measurements (Goniometer LK-1, NPK Open Science, Krasnogorsk, Russia) using the sessile drop method and “DropShape” analysis software (version 1, Laboratory of Mathematical Methods of Image Processing, Lomonosov Moscow State University, Moscow, Russia). Thermal stability was evaluated by thermogravimetric analysis (TGA, Netzsch TG 209 F1 Libra, Leuna, Germany) under argon with a heating rate of 10 °C min^−1^ from 30 to 550 °C.

### 3.6. DFT Calculations

The nature of noncovalent interactions between NaAlg and ZnO was examined using quantum chemical calculations. All computations were performed with the Gaussian 16 W, Revision A.03 software package [65] using density functional theory (DFT). The B3LYP functional, augmented with Grimme’s D3 empirical dispersion correction and Becke–Johnson damping (D3(BJ)), was employed together with the aug-cc-pVDZ basis set [66,67,68]. Geometry optimizations were conducted for the singlet ground state without symmetry constraints, followed by vibrational frequency calculations. The thermodynamic stability of the formed complexes was assessed by calculating standard-state (298.15 K, 1 atm) Gibbs free energies.

To characterize the intermolecular binding, bond order analysis and a topological study of the electron density distribution were carried out using Bader’s Quantum Theory of Atoms in Molecules (QTAIM) within the Multiwfn 2026.2.2 program [69,70]. The analysis was complemented by visualizing noncovalent interaction (NCI) regions. These visualizations, generated with the VMD software, version 1.9.4a53 [71], are based on reduced density gradient isosurfaces (value = 0.5 (a.u.)) mapped onto a color scale representing the sign(λ_2_)ρ function in the range of [−0.04, 0.02] e bohr^−3^ [72].

## 4. Conclusions

This study presents the first comprehensive investigation into the development and application of ZnO nanoplates as a modifier for NaAlg-based MMMs for the pervaporation dehydration of IPA. An optimal modifier loading of 5 wt.% was identified, resulting in a 32% increase in permeation flux for the separation of a 30 wt.% water feed mixture. These performance enhancements were attributed to significant morphological changes in the membranes (confirmed by SEM), which facilitated the formation of new transport pathways at the polymer–modifier interface, increased the effective surface area, and enhanced surface hydrophilicity (confirmed by AFM and contact angle measurements). The retention of exceptionally high water selectivity (>99.99 wt.% in the permeate) for modified membranes was likely associated with a specific interaction between the modifier and the polymer matrix (confirmed by FTIR, NMR, and DFT investigation). This interaction increased the composite’s crystallinity and contributed to its improved thermal stability (confirmed by TGA). A further increase in the modifier content to 7 wt.% led to agglomeration and a consequent deterioration of transport properties compared to the NaAlg-5 membrane.

The NaAlg-5^CL^ membrane cross-linked with CaCl_2_ was evaluated for the separation of mixtures with water content up to 90 wt.%. Cross-linking induced an additional improvement in transport properties due to polymer matrix reorganization (confirmed by XRD, FTIR, NMR, and TGA), which reduced crystallinity and facilitated the diffusion of feed components. The cross-linked NaAlg-5^CL^ membrane exhibited the best overall performance among the dense membranes, with permeation fluxes ranging from 0.192 to 0.794 kg m^−2^h^−1^ for separating 12–90 wt.% water/IPA mixtures.

To further enhance transport properties, supported membranes were fabricated. The deposition of a thin selective composite layer onto a CA substrate led to an increase in permeation flux, although with a slight decrease in water content in the permeate. The permeation flux of cross-linked supported NaAlg-5^CL^/CA membrane increased by 2 or more times compared to the dense NaAlg membrane when dehydrating IPA (12–30 wt.% water). A comparative analysis of the transport characteristics against literature analogues demonstrated the high promise of using cost-effective ZnO nanoplates as a modifier for creating high-performance MMMs.

## Figures and Tables

**Table 1:** Liquid uptake and water contact angle for developed cross-linked dense membranes.

Membrane	LU, %			Contact Angle of Water, °
	H_2_O	IPA	H_2_O/IPA (50:50 wt.%)	
NaAlg^CL^	73	1	35	69
NaAlg-5^CL^	76	2	39	53

**Table 2:** Change in Gibbs free energy (kJ mol^−1^) for the formation of the most stable associates (B3LYP-D3(BJ)/aug-cc-pVDZ level).

B3LYP-D3(BJ)/aug-cc-pVDZ			
∆G_min_, kJ mol^−1^			
	ZnO	H_2_O	IPA
**^M^** **NaAlg**	−277.9	−37.3	−43.4
**^G^** **NaAlg**	−256.1	−34.9	−37.8
**ZnO**	~	−59.7	−83.6
**H_2_O**		~	10.4

**Table 3:** Comparison of the transport characteristics of the NaAlg-based membranes during IPA dehydration of azeotropic mixture.

Membrane *	Thickness, μm	Water Content in the Feed, wt.%	Temp., °C	Permeation Flux, g m^−2^h^−1^	Separation Factor, β	Ref.
NaAlg-5^CL^	30	12	22	193	73,326	This study
NaAlg-5^CL^/CA	0.25	12	22	279	73,326	This study
PERVAP™ 1201	-	12	22	28	73,326	[9]
NaAlg-3 porous CuO^CL^	37.5	10	25	600	32,828	[13]
NaAlg-0.25 TiO_2_	-	10	30	40	∞	[59]
NaAlg-40 TiO_2_^CL(PSSA-co-MA)^	40	10	30	186.1	24,092	[60]
NaAlg-30 NaY	40	10	30	232	272	[24]
NaAlg-10 Fe-SBA-15	50	10	30	35	∞	[61]
NaAlg-20 Al-MCM-41	60	10	30	214	∞	[62]
NaAlg-20 AlPO_4_-5	50	12.6	30	77	69,000	[63]
NaAlg-10 charcoal	60	12.5	30	410	2326	[64]

* Zeolites: NaY; Molecular sieves: MCM-41, AlPO_4_-5, Fe-SBA-15; poly(styrene sulfonic acid-co-maleic acid) (PSSA-co-MA).

**Table 4:** Developed dense and supported membranes based on NaAlg and NaAlg-ZnO composites.

Membrane	Type	Content of ZnO, wt.%	CaCl_2_ Cross-Linking	Porous Support
NaAlg	Dense	0	−	−
NaAlg-3	Dense	3	−	−
NaAlg-5	Dense	5	−	−
NaAlg-7	Dense	7	−	−
NaAlg^CL^	Dense	0	+	−
NaAlg-5^CL^	Dense	5	+	−
NaAlg^CL^/CA	Supported	0	+	+
NaAlg-5^CL^/CA	Supported	5	+	+

## Data Availability

Data are contained within the article and Supplementary Materials.

## Supplementary Materials

The following supporting information can be downloaded at: https://www.mdpi.com/article/10.3390/molecules31081300/s1, Table S1. Atomic coordinates (x, y, z) for the optimized structures.; Table S2. Calculated changes in thermodynamic functions for the 1:1 associate formation.; Table S3. Distance (d), ratio of d to the sum of the van der Waals radii of the interacting atoms ration of the distance to the sum of VW radiuses, WBI, and FBO.; Figure S1. Cross-sectional SEM micrographs of the dense membranes at ×3000 magnification.; Figure S2. Cross-sectional SEM micrographs of the supported membranes at ×800, ×8000, and ×40,000 magnifications.; Figure S3. Dependence of liquid uptake on the water content in the H_2_O/IPA mixture for cross-linked NaAlg^CL^ and NaAlg-5^CL^ membranes.
