# Supplementary material for: Pervaporation Mixed Matrix Membranes from Sodium Alginate/ZnO for Isopropanol Dehydration
Source: Molecules. 2026 Apr 16;31(8):1300. doi: 10.3390/molecules31081300 (PMC13119433; doi:10.3390/molecules31081300)
Supplement: Supplementary file 1 [file molecules-31-01300-s001.zip › molecules-4211498-supplementary.pdf]

Pervaporation Mixed Matrix Membranes From Sodium Alginate/ZnO for Isopropanol Dehydration

Roman Dubovenko[1], Mariia Dmitrenko[1,*], Anna Mikulan[1], Olga Mikhailovskaya[1], Anna Kuzminova[1], Aleksandra Koroleva[1], Anton Mazur[1], Rongxin Su[2], and Anastasia Penkova[1,*]

[1] St. Petersburg State University, 7/9 Universitetskaya nab., St. Petersburg 199034, Russia; r.dubovenko@spbu.ru (R.D.), st097675@student.spbu.ru (A. M.), st113220@student.spbu.ru (O.M.), a.kuzminova@spbu.ru (A.K.), aleksandra.koroleva@spbu.ru (A.K.), a.mazur@spbu.ru (A.M.)

[2] State Key Laboratory of Chemical Engineering, School of Chemical Engineering and Technology, Tianjin University, Tianjin 300072, China; surx@tju.edu.cn

* Correspondence: m.dmitrienko@spbu.ru (M.D.), a.penkova@spbu.ru (A.P.), tel.: +7(812)363-60-00, additional code 3367

Table S1. Atomic coordinates (x, y, z) for the optimized structures.

Atom	*X*	*Y*	*Z*	*Atom*	*X*	*Y*	*Z*
^{M}*NaAlg*				**H**	4.743446	0.247286	0.185168
**C**	-0.598720	-1.651494	-0.068694	**H**	3.174292	-0.471804	1.652981
**C**	0.541152	-1.317189	0.890889	**H**	0.010671	-1.849625	-1.223288
**C**	1.738362	-0.746511	0.099471	**Na**	-3.638075	-1.774987	-0.176793
**C**	1.322618	0.211773	-1.068255	**C**	-0.665340	-0.250706	0.497092
**O**	-0.068501	0.501006	-1.186190	**O**	-1.083882	0.961862	0.434609
**C**	-1.119404	-0.403079	-0.780567	**O**	-1.370164	-1.267825	0.270591
**O**	1.917049	1.487635	-0.859183	**H**	0.752471	-0.847845	1.933411
**O**	2.590004	-0.053672	1.014725	^{G}*NaAlg···ZnO-2*			
**O**	0.187242	-0.422099	1.936733	**O**	3.324994	-1.369230	0.355597
**O**	-0.024794	-2.589978	-0.992515	**Zn**	1.636659	-0.935920	0.279314
**C**	-2.130227	0.360880	0.134423	**C**	-1.908010	1.493869	0.349003
**O**	-3.291874	-0.033104	0.108160	**C**	-2.866699	0.300702	0.404399
**O**	-1.611653	1.298512	0.858318	**C**	-2.502582	-0.729798	-0.693897
**H**	-1.436221	-2.104376	0.479276	**C**	-0.979966	-0.778874	-0.915631
**H**	0.870656	-2.242605	1.378031	**O**	-0.311552	-0.400320	0.365325
**H**	2.315238	-1.567327	-0.336206	**C**	-0.467485	1.029585	0.663555
**H**	1.665755	-0.187101	-2.032284	**O**	-0.516721	-2.027001	-1.239277
**H**	-1.660868	-0.707142	-1.685631	**O**	-2.968960	-2.027178	-0.343184
**H**	2.515792	1.342309	-0.093921	**O**	-2.876146	-0.325691	1.691652
**H**	2.013998	0.133883	1.783382	**O**	-2.036919	2.044014	-0.950181
**H**	-0.458979	0.263166	1.623844	**H**	-2.197703	2.227825	1.115300
**H**	-0.725595	-2.921164	-1.566240	**H**	-3.891384	0.651842	0.248693
**Na**	-0.109295	2.523425	-0.166748	**H**	-2.968689	-0.444101	-1.643114
^{G}*NaAlg*				**H**	-0.646332	-0.052233	-1.663284
**C**	0.219272	-1.505413	0.033161	**H**	-1.168847	-2.664198	-0.888900
**C**	1.717935	-1.234236	-0.099979	**H**	-3.171475	-2.001769	0.607432
**C**	1.958916	0.109504	-0.819622	**H**	-1.970662	-0.580528	1.918385
**C**	0.938351	1.173133	-0.320844	**H**	-1.355621	2.742323	-1.002959
**O**	0.437332	0.772037	0.978770	**Na**	3.923928	0.603593	-0.219286
**C**	-0.424425	-0.357981	0.876227	**C**	0.642478	1.864759	-0.028663
**O**	1.515669	2.421758	-0.210263	**O**	1.727778	1.225780	-0.245054
**O**	3.296809	0.549443	-0.575841	**O**	0.393677	3.051713	-0.269311
**O**	2.359258	-1.218656	1.182961	**H**	-0.287889	1.100766	1.744039

**O**	-0.289802	-1.592756	-1.293071	*^{G}NaAlg···ZnO-3*			
**H**	0.071008	-2.459503	0.560391	**O**	3.365898	-0.507196	-0.559593
**H**	2.191661	-2.048751	-0.658440	**Zn**	1.754346	-1.207880	-0.610103
**H**	1.846133	-0.014326	-1.901818	**C**	-0.697809	1.554488	-0.645261
**H**	0.076380	1.240342	-0.997933	**C**	0.786515	1.787967	-0.357700
**H**	2.474506	2.268866	-0.138255	**C**	1.120560	1.245929	1.060350
**H**	3.584837	0.094118	0.233149	**C**	0.309752	-0.053499	1.375195
**H**	1.934546	-0.504058	1.686375	**O**	-0.042654	-0.751022	0.085700
**H**	-1.254947	-1.666537	-1.187650	**C**	-0.981874	0.025830	-0.708761
**Na**	-4.173141	0.508208	-0.357806	**O**	1.065001	-0.940269	2.095050
**C**	-1.843338	-0.001214	0.391981	**O**	2.491962	1.040169	1.306871
**O**	-2.224993	1.194668	0.400284	**O**	1.471069	1.158159	-1.436044
**O**	-2.578045	-0.987493	0.025529	**O**	-1.449465	2.215255	0.364839
**H**	-0.549651	-0.715196	1.907549	**H**	-0.937460	1.969576	-1.633996
ZnO				**H**	0.994189	2.867269	-0.364281
**O**	0.000000	0.000000	-1.351396	**H**	0.772123	2.002531	1.774436
**Zn**	0.000000	0.000000	0.360372	**H**	-0.660264	0.132869	1.845735
H_2O				**H**	1.960386	-0.525365	2.134532
**O**	0.000000	0.000000	0.117813	**H**	2.939562	0.446024	0.565435
**H**	0.000000	0.764149	-0.471250	**H**	2.399803	0.876761	-1.195570
**H**	0.000000	-0.764149	-0.471250	**H**	-2.380053	2.069977	0.129753
IPA				**Na**	-4.810223	-0.856778	0.238166
**O**	0.000229	1.418129	0.022142	**C**	-2.426830	-0.323788	-0.319651
**H**	0.001130	1.458813	0.987353	**O**	-2.659883	-1.374512	0.327755
**C**	-0.000022	0.036390	-0.370852	**O**	-3.321067	0.497759	-0.716587
**H**	-0.000040	0.080311	-1.469526	**H**	-0.834988	-0.307011	-1.741745
**C**	1.270375	-0.668381	0.099571	*^{G}NaAlg···ZnO-4*			
**H**	1.302061	-0.715429	1.199499	**O**	-4.036650	-2.224460	-0.346088
**H**	2.159688	-0.129207	-0.250341	**Zn**	-3.000524	-0.890597	-0.278914
**H**	1.310992	-1.698689	-0.281857	**C**	0.154190	-0.481214	0.967074
**C**	-1.270664	-0.667902	0.099646	**C**	-0.806089	0.708945	0.979448
**H**	-1.311744	-1.698164	-0.281865	**C**	-0.039965	2.029149	0.841512
**H**	-1.302278	-0.714987	1.199570	**C**	1.053297	1.876457	-0.262644
**H**	-2.159770	-0.128316	-0.250153	**O**	0.692498	0.744953	-1.112584
^{M}NaAlg···ZnO-1				**C**	0.882614	-0.496720	-0.417078
**O**	3.380909	-0.868301	-0.165828	**O**	1.165420	3.003018	-1.040828
**Zn**	2.482752	0.589174	0.052697	**O**	-0.923871	3.108391	0.539818
**C**	-1.998012	1.009455	-0.945027	**O**	-1.712050	0.611312	-0.178364
**C**	-2.769756	0.214557	0.109194	**O**	1.016933	-0.287382	2.072790
**C**	-2.325594	-1.281757	0.080106	**H**	-0.416671	-1.416449	1.081125
**C**	-0.903478	-1.453589	-0.499187	**H**	-1.447799	0.704391	1.864409
**O**	-0.076938	-0.341141	-0.140823	**H**	0.441924	2.264890	1.794987
**C**	-0.479047	0.916414	-0.719230	**H**	2.029976	1.640596	0.176688
**O**	-0.299308	-2.590321	0.063165	**H**	0.354554	3.523806	-0.908216
**O**	-2.340785	-1.818315	1.397572	**H**	-1.711040	2.753404	0.102756
**O**	-2.570926	0.724504	1.419831	**H**	-1.094013	0.672100	-0.951147
**O**	-2.372452	0.431248	-2.204303	**H**	1.700893	-0.977683	1.982722
**C**	0.075039	1.979694	0.258563	**Na**	4.717418	-1.767322	-0.180544
**O**	1.366359	2.059653	0.341809	**C**	2.360217	-0.914703	-0.306343
**O**	-0.707872	2.676825	0.906184	**O**	3.214666	-0.386308	-1.055197
**H**	-2.292196	2.065322	-0.897941	**O**	2.612162	-1.830085	0.552137

**H**	-3.839863	0.268494	-0.128521	**H**	0.407840	-1.253605	-1.055216
**H**	-3.019120	-1.870456	-0.531175	***^{G}NaAlg···ZnO-5***			
**H**	-0.918326	-1.564774	-1.590821	**O**	-3.421951	1.286738	0.655108
**H**	0.021527	1.034897	-1.696411	**Zn**	-3.058871	-0.243638	-0.096973
**H**	-0.770912	-2.705983	0.916420	**C**	0.892935	-1.698608	-0.136207
**H**	-2.276727	-1.039260	1.985327	**C**	-0.412098	-1.157782	0.466570
**H**	-1.985600	1.513436	1.388680	**C**	-0.213571	0.315573	0.874117
**H**	-2.127435	1.035160	-2.914441	**C**	-0.102492	1.161237	-0.424340
**Na**	2.009026	-2.446720	-0.069036	**O**	0.504939	0.331511	-1.454732
^{M}NaAlg···ZnO-2				**C**	1.525857	-0.569688	-1.033941
**O**	-3.664675	0.833578	0.051499	**O**	-1.327902	1.511731	-1.000944
**Zn**	-2.762576	-0.638460	0.119286	**O**	-1.084106	0.824144	1.846310
**C**	2.363980	-1.342353	-0.021793	**O**	-1.481561	-1.340260	-0.568527
**C**	2.783791	-0.019752	0.625713	**O**	1.699831	-2.142239	0.931379
**C**	2.472040	1.189236	-0.288503	**H**	0.634221	-2.548092	-0.789954
**C**	1.130397	1.109751	-1.067296	**H**	-0.722507	-1.772792	1.314437
**O**	0.791272	-0.169215	-1.522946	**H**	0.779345	0.318146	1.347601
**C**	0.966411	-1.299387	-0.644593	**H**	0.528398	2.043523	-0.268539
**O**	0.079067	1.675365	-0.263708	**H**	-2.036280	1.752831	-0.325056
**O**	2.452621	2.368576	0.518690	**H**	-2.000592	1.064247	1.481715
**O**	2.234531	0.182545	1.917256	**H**	-1.117896	-0.864506	-1.348319
**O**	3.352366	-1.584610	-1.026944	**H**	2.622158	-1.882158	0.723828
**C**	-0.212411	-1.352021	0.346435	**Na**	4.814999	1.114379	0.620809
**O**	-1.310807	-1.783762	-0.197400	**C**	2.769288	0.103505	-0.430510
**O**	-0.113195	-0.955301	1.513171	**O**	2.856139	1.354514	-0.376747
**H**	2.384656	-2.129959	0.748192	**O**	3.688677	-0.695512	-0.030136
**H**	3.872384	-0.057824	0.756423	**H**	1.882237	-1.030664	-1.963245
**H**	3.269561	1.279593	-1.035299	***^{G}NaAlg···H_2O-1***			
**H**	1.203314	1.700610	-1.990125	**H**	-5.349947	-0.644289	-0.671742
**H**	0.848803	-2.158435	-1.316717	**O**	-4.583959	-0.879160	-0.136867
**H**	0.571116	2.214578	0.386793	**H**	-3.840012	-1.014498	-0.761116
**H**	2.444628	2.035065	1.437829	**C**	0.019035	-1.395489	0.066437
**H**	1.316090	-0.186027	1.922136	**C**	-1.439164	-0.950329	0.194165
**H**	3.105193	-2.363368	-1.539660	**C**	-1.507496	0.436678	0.873951
**Na**	-2.157559	2.233574	-0.414987	**C**	-0.386176	1.357197	0.307158
^{M}NaAlg···ZnO-3				**O**	0.030934	0.844693	-0.983036
**O**	-2.583149	0.751674	0.420530	**C**	0.763198	-0.369488	-0.846723
**Zn**	-1.467597	1.018658	-0.904951	**O**	-0.809816	2.660374	0.149415
**C**	1.888827	-0.820055	-0.852822	**O**	-2.750293	1.096512	0.662799
**C**	0.473232	-0.900462	-1.450980	**O**	-2.053764	-0.949893	-1.106303
**C**	-0.414085	-1.768193	-0.472686	**O**	0.544264	-1.475769	1.385992
**C**	0.048446	-1.579515	0.995748	**H**	0.048321	-2.384329	-0.414776
**O**	0.576759	-0.247533	1.176398	**H**	-1.997336	-1.684411	0.785786
**C**	1.858861	-0.109204	0.530501	**H**	-1.336228	0.296512	1.950138
**O**	-1.013768	-1.706987	1.900969	**H**	0.494670	1.352255	0.961985
**O**	-1.818259	-1.619418	-0.595869	**H**	-1.778101	2.623428	0.063358
**O**	0.067659	0.434886	-1.745213	**H**	-3.474699	0.457396	0.518850
**O**	2.376941	-2.160837	-0.738980	**H**	-1.681058	-0.185896	-1.577014
**C**	2.152543	1.401492	0.498846	**H**	1.492783	-1.660865	1.267011
**O**	2.839405	1.915814	1.354653	**Na**	4.615795	0.124955	0.263798
**O**	1.603863	2.095240	-0.497755	**C**	2.225877	-0.153369	-0.413221

**H**	2.526272	-0.245370	-1.539913	**O**	2.742630	0.987552	-0.499806
**H**	0.527084	-1.449206	-2.401053	**O**	2.852634	-1.196085	-0.003904
**H**	-0.204212	-2.809881	-0.743497	**H**	0.815168	-0.786786	-1.861187
**H**	0.842459	-2.289334	1.260597	***^{G}NaAlg···H_2O-2***			
**H**	2.624237	-0.562788	1.176812	**H**	-1.935172	-1.980248	2.879440
**H**	-1.802690	-1.895941	1.356077	**O**	-2.173740	-1.489048	2.086650
**H**	-2.189543	-0.772612	-0.150868	**H**	-1.463931	-1.646455	1.428685
**H**	1.054131	1.483309	-1.087549	**C**	0.024265	1.478585	0.377847
**H**	3.327376	-2.140302	-0.578539	**C**	-1.469288	1.458150	0.051811
**Na**	-1.374524	0.643210	2.196996	**C**	-1.751611	0.484610	-1.124798
^{M}NaAlg···H_2O-1				**C**	-0.780980	-0.726949	-1.120381
**C**	1.264469	1.545963	-0.077182	**O**	-0.319933	-0.976724	0.230734
**C**	0.051892	1.464858	0.847407	**C**	0.525796	0.051767	0.737894
**C**	-1.189172	1.152762	0.012743	**O**	-1.401886	-1.871357	-1.577461
**C**	-1.028063	-0.101942	-0.897704	**O**	-3.099511	0.024864	-1.061319
**O**	0.296680	-0.455852	-1.250756	**O**	-2.284914	1.172040	1.182873
**C**	1.506596	0.218640	-0.797134	**O**	0.664065	2.015478	-0.778147
**O**	-1.572134	-1.282687	-0.291655	**H**	0.192002	2.135649	1.243274
**O**	-2.303861	1.010418	0.895760	**H**	-1.758360	2.469841	-0.253531
**O**	0.175459	0.531611	1.910995	**H**	-1.626085	1.013855	-2.076621
**O**	0.967532	2.611894	-0.991612	**H**	0.105295	-0.527398	-1.736895
**C**	2.382646	-0.701840	0.107655	**H**	-2.354801	-1.733459	-1.424041
**O**	3.597580	-0.542423	0.016186	**H**	-3.404836	0.208720	-0.155704
**O**	1.733455	-1.493842	0.892242	**H**	-2.089164	0.300860	1.581507
**H**	2.165740	1.779124	0.506403	**H**	1.614008	1.996297	-0.570608
**H**	-0.097624	2.448478	1.310329	**Na**	4.415734	-0.468484	-0.229888
**H**	-1.405159	2.006464	-0.636048	**C**	2.002699	-0.161963	0.348535
**H**	-1.572615	0.066640	-1.838187	**O**	2.382303	-1.292071	-0.046297
**H**	2.081297	0.409490	-1.710306	**O**	2.778191	0.850321	0.482571
**H**	-2.551107	-1.224700	-0.322925	**H**	0.486094	-0.057563	1.830968
**H**	-1.944344	0.542278	1.669919	***^{G}NaAlg···H_2O-3***			
**H**	0.687055	-0.264448	1.625312	**H**	-4.288739	2.588836	-0.714698
**H**	1.757007	2.788829	-1.516129	**O**	-4.084131	1.691836	-0.435195
**Na**	0.176269	-2.603500	-0.088943	**H**	-3.211629	1.706691	0.011757
**H**	-5.010070	-0.736846	0.028814	**C**	0.884559	-1.576304	-0.125468
**O**	-4.170165	-0.486388	-0.370054	**C**	2.288599	-0.990482	-0.282281
**H**	-3.775721	0.210708	0.197644	**C**	2.207264	0.447897	-0.837427
^{M}NaAlg···H_2O-2				**C**	1.020992	1.205820	-0.176781
**C**	1.364262	1.216359	0.601793	**O**	0.704818	0.573589	1.087115
**C**	-0.041522	1.132117	1.205297	**C**	0.085433	-0.695357	0.886828
**C**	-1.062886	1.602146	0.151042	**O**	1.324539	2.533316	0.050149
**C**	-0.750514	1.017817	-1.267267	**O**	3.435852	1.132103	-0.583856
**O**	0.239977	-0.009605	-1.231530	**O**	2.996870	-0.990670	0.964666
**C**	1.501546	0.243009	-0.586945	**O**	0.318097	-1.602500	-1.430431
**O**	-1.880988	0.378202	-1.799825	**H**	0.961554	-2.597429	0.276762
**O**	-2.392814	1.268194	0.550603	**H**	2.877494	-1.620300	-0.957582
**O**	-0.348657	-0.183646	1.673721	**H**	2.059627	0.428152	-1.922293
**O**	1.516778	2.585172	0.202338	**H**	0.122987	1.151481	-0.807332
**C**	2.041814	-1.128301	-0.083414	**H**	2.296564	2.585761	0.072994
**O**	3.254552	-1.305994	-0.121840	**H**	3.860791	0.656663	0.149706
**O**	1.125104	-1.915018	0.382386	**H**	2.472153	-0.443933	1.572748

**H**	2.115734	0.956097	1.360728	**H**	-0.608497	-1.871264	-1.297156
**H**	-0.105101	1.805594	2.068623	**Na**	-3.896661	-0.531227	-0.170002
**H**	-1.023042	2.693190	0.085470	**C**	-1.410078	-0.583687	0.530396
**H**	-0.408617	1.811137	-1.948216	**O**	-2.022101	0.486235	0.775505
**H**	2.211092	0.653668	-1.317575	**O**	-1.955743	-1.624364	0.027487
**H**	-2.592417	0.532518	-1.147566	**H**	0.114729	-1.184840	1.869982
**H**	-2.341837	0.467416	1.092749	***^{G}NaAlg···IPA-1***			
**H**	0.255810	-0.867812	1.238635	**O**	-3.670499	0.629456	-0.558671
**H**	2.427792	2.717676	-0.084796	**H**	-3.011952	0.014563	-0.936759
**Na**	-0.785433	-1.979313	-0.846859	**C**	0.795349	-1.302464	0.038808
**O**	-2.249836	-1.994865	0.945356	**C**	-0.573712	-0.650651	0.255914
**H**	-2.652416	-2.646080	1.529559	**C**	-0.391297	0.686040	0.995869
**H**	-1.676368	-1.429385	1.505145	**C**	0.766931	1.502259	0.328252
^{M}NaAlg···H2O-3				**O**	1.062860	0.940812	-0.977798
**C**	-1.269135	1.567054	0.088833	**C**	1.646219	-0.354593	-0.871575
**C**	-1.614535	0.419200	1.036458	**O**	0.424603	2.826561	0.165084
**C**	-1.987839	-0.843890	0.221561	**O**	-1.572639	1.475243	1.060846
**C**	-1.132161	-1.016163	-1.075207	**O**	-1.229026	-0.490368	-1.014028
**O**	0.002387	-0.152415	-1.159700	**O**	1.360099	-1.528053	1.323424
**C**	-0.050773	1.233465	-0.779662	**H**	0.651428	-2.259208	-0.485402
**O**	-0.563354	-2.310110	-1.099470	**H**	-1.212959	-1.318094	0.842884
**O**	-1.825544	-1.984891	1.065036	**H**	-0.093807	0.457460	2.025714
**O**	-0.585708	0.104139	1.964070	**H**	1.690884	1.433029	0.918518
**O**	-2.450970	1.743905	-0.707130	**H**	-0.539443	2.866825	0.313990
**C**	1.275649	1.568355	-0.034861	**H**	-2.279501	1.165268	0.454527
**O**	1.739701	2.693224	-0.179726	**H**	-0.704180	0.173457	-1.495554
**O**	1.733618	0.600073	0.695910	**H**	2.273936	-1.817866	1.149439
**H**	-1.047948	2.477562	0.663185	**Na**	5.558005	-0.361323	0.138564
**H**	-2.488772	0.708968	1.631189	**C**	3.133551	-0.327388	-0.471643
**H**	-3.039868	-0.795777	-0.075693	**O**	3.781450	0.745790	-0.537377
**H**	-1.748962	-0.869174	-1.972071	**O**	3.641202	-1.451593	-0.115969
**H**	-0.081131	1.847033	-1.690106	**H**	1.629202	-0.755807	-1.893680
**H**	-0.907307	-2.735245	-0.284138	**C**	-4.749199	-0.116845	0.029108
**H**	-1.231602	-1.675083	1.778673	**H**	-5.382813	0.649236	0.497806
**H**	0.313215	0.231881	1.562069	**C**	-4.238917	-1.069286	1.109284
**H**	-2.335964	2.527483	-1.257374	**H**	-3.651553	-0.525562	1.859604
**Na**	1.702494	-1.452756	-0.475188	**H**	-3.606749	-1.852914	0.665590
**H**	4.711096	-0.870639	0.377381	**H**	-5.079790	-1.562750	1.615557
**O**	3.758607	-0.980393	0.312208	**C**	-5.548904	-0.836220	-1.053782
**H**	3.332678	-0.143093	0.621645	**H**	-4.927293	-1.598076	-1.547895
^{M}NaAlg···IPA-1				**H**	-6.424735	-1.339892	-0.621009
**C**	-2.008914	1.640516	-0.002502	**H**	-5.893249	-0.123001	-1.813035
**C**	-2.566826	0.542575	0.902019	***^{G}NaAlg···IPA-2***			
**C**	-2.914870	-0.710333	0.060166	**O**	-2.009387	1.135022	-1.116293
**C**	-1.907781	-0.976547	-1.105954	**H**	-1.444167	1.097903	-0.317442
**O**	-0.719119	-0.185401	-1.071133	**C**	1.070637	-1.212084	-1.136816
**C**	-0.715630	1.203586	-0.697932	**C**	-0.190653	-1.996667	-0.773824
**O**	-1.423648	-2.302709	-1.019922	**C**	-0.407573	-1.993268	0.764076
**O**	-2.951617	-1.838366	0.934770	**C**	0.063887	-0.665162	1.415637
**O**	-1.706598	0.178399	1.970078	**O**	0.028195	0.399979	0.440934
**O**	-3.056259	1.886641	-0.953469	**C**	0.993210	0.245767	-0.596891

**C**	0.532947	1.428395	0.204768	**O**	-0.756355	-0.306807	2.469896
**O**	1.171774	2.466323	0.051270	**O**	-1.789367	-2.208836	1.047433
**O**	0.772607	0.466416	1.036938	**O**	-1.356764	-1.557681	-1.462651
**H**	-1.799945	2.544068	0.587797	**O**	2.152042	-1.954373	-0.575755
**H**	-3.493194	0.908795	1.360322	**H**	1.164964	-1.174498	-2.231621
**H**	-3.910067	-0.595799	-0.379993	**H**	-0.041108	-3.031718	-1.100670
**H**	-2.392439	-0.827468	-2.080071	**H**	0.157744	-2.815301	1.217788
**H**	-0.575785	1.807132	-1.604769	**H**	1.098754	-0.746028	1.774381
**H**	-1.903501	-2.679691	-0.250213	**H**	-1.591664	-0.786901	2.321451
**H**	-2.441505	-1.550630	1.718988	**H**	-2.254257	-2.014588	0.213501
**H**	-0.752162	0.228056	1.692043	**H**	-1.511843	-0.591013	-1.381124
**H**	-2.802386	2.636373	-1.504413	**H**	2.946334	-1.428175	-0.771956
**Na**	0.797901	-1.594668	-0.177693	**Na**	4.636059	1.697742	0.371750
**C**	4.930486	0.212071	0.812244	**C**	2.376660	0.792450	-0.194605
**H**	4.406321	1.126730	1.124938	**O**	2.483357	1.543072	0.806523
**H**	5.172826	-0.378904	1.703931	**O**	3.358594	0.455029	-0.947606
**H**	5.870008	0.506636	0.323079	**H**	0.639547	0.894566	-1.409525
**C**	4.058350	-0.594894	-0.142661	**C**	-3.338610	1.540144	-0.748062
**H**	4.593022	-1.505646	-0.448653	**H**	-3.856441	1.683723	-1.707038
**O**	2.887198	-1.081835	0.546872	**C**	-3.295824	2.865193	0.007735
**H**	2.412537	-0.312233	0.938387	**H**	-2.778542	3.629381	-0.585905
**C**	3.655216	0.204690	-1.380239	**H**	-2.762103	2.744098	0.962089
**H**	4.542842	0.516739	-1.945655	**H**	-4.312353	3.219966	0.227356
**H**	3.029041	-0.403673	-2.050671	**C**	-4.046955	0.440954	0.041544
**H**	3.094907	1.104761	-1.093122	**H**	-3.529298	0.255919	0.993282
^{M}NaAlg···IPA-2				**H**	-5.081532	0.733338	0.267797
**C**	2.209516	-0.089837	-1.167788	**H**	-4.071883	-0.493042	-0.534229
**C**	0.880934	-0.826605	-1.371125	***^{G}NaAlg···IPA-3***			
**C**	0.765229	-1.944623	-0.307704	**H**	-2.424985	0.149049	-0.461447
**C**	1.308303	-1.480107	1.081205	**O**	-3.171113	0.219124	0.169155
**O**	1.375854	-0.053149	1.162386	**C**	2.205464	1.303093	0.374632
**C**	2.229022	0.611986	0.210270	**C**	3.088493	0.053350	0.441209
**O**	0.446209	-1.864849	2.111577	**C**	2.212327	-1.191570	0.709432
**O**	-0.569098	-2.435090	-0.193153	**C**	0.888851	-1.078880	-0.093145
**O**	-0.224339	0.078783	-1.330195	**O**	1.107229	-0.218814	-1.233063
**O**	3.223688	-1.096799	-1.280901	**C**	1.207112	1.146313	-0.815037
**C**	1.721246	2.075905	0.066591	**O**	0.445678	-2.312086	-0.533813
**O**	2.563162	2.951015	-0.106790	**O**	2.915814	-2.375978	0.331730
**O**	0.432119	2.191556	0.090371	**O**	3.840450	-0.131186	-0.765416
**H**	2.339611	0.668234	-1.953160	**O**	1.576588	1.402673	1.645389
**H**	0.873513	-1.295228	-2.362144	**H**	2.837693	2.185265	0.189097
**H**	1.368680	-2.795634	-0.637272	**H**	3.830581	0.168816	1.238051
**H**	2.315158	-1.884223	1.259761	**H**	1.980354	-1.270911	1.776478
**H**	3.257809	0.633042	0.594500	**H**	0.101959	-0.624663	0.524979
**H**	-0.292953	-2.326099	1.671254	**H**	1.232994	-2.884093	-0.564795
**H**	-1.183003	-1.685485	-0.243061	**H**	3.606689	-2.090576	-0.290000
**H**	0.028855	0.932739	-0.859470	**H**	3.188271	-0.214632	-1.480451
**H**	4.084915	-0.663313	-1.267613	**H**	0.874913	2.072776	1.525609
**Na**	-0.586404	0.940878	1.669354	**Na**	-2.562688	2.384407	0.203851
**C**	-4.123658	-0.837339	-0.906196	**C**	-0.190807	1.754605	-0.565003
**H**	-3.583180	-0.742683	-1.859634	**O**	-1.156393	1.295433	-1.229672

**H**	-3.934985	-1.836590	-0.493317	**O**	-0.300444	2.696973	0.288257
**H**	-5.197499	-0.745632	-1.115622	**H**	1.611470	1.681365	-1.684881
**C**	-3.674676	0.244759	0.069228	**C**	-3.688412	-1.097000	0.457818
**H**	-4.198990	0.116657	1.026384	**H**	-4.581495	-0.908175	1.070557
**O**	-2.277818	0.061078	0.397791	**C**	-4.093777	-1.792809	-0.837286
**H**	-1.742781	0.118288	-0.425695	**H**	-3.211158	-1.958196	-1.472233
**C**	-3.906913	1.655515	-0.460091	**H**	-4.818829	-1.186449	-1.395022
**H**	-4.974826	1.823522	-0.652361	**H**	-4.545335	-2.771082	-0.623801
**H**	-3.561466	2.406975	0.261393	**C**	-2.676280	-1.904473	1.264239
**H**	-3.360645	1.807498	-1.401853	**H**	-3.115708	-2.863033	1.572815
^{M}NaAlg···IPA-3				**H**	-1.781795	-2.122896	0.664550
**C**	-0.054150	-0.949073	1.139062	**H**	-2.376346	-1.355934	2.166436
**C**	-0.483903	-1.432246	-0.244915	***ZnO···H_2O-1***			
**C**	-2.023158	-1.487679	-0.325510	**O**	1.848840	-0.003660	0.036722
**C**	-2.734227	-0.306969	0.420971	**Zn**	0.159322	0.001770	-0.013751
**O**	-1.884295	0.713935	0.940756	**O**	-1.862628	-0.002105	-0.053012
**C**	-0.536025	0.476802	1.406093	**H**	-2.333424	-0.784365	0.269626
**O**	-3.572000	0.387094	-0.495472	**H**	-2.335926	0.777401	0.273215
**O**	-2.410287	-1.494822	-1.701989	***ZnO···IPA-1***			
**O**	0.026885	-0.645187	-1.323351	**O**	2.876502	0.310738	-0.275199
**O**	-0.665331	-1.883034	2.044901	**Zn**	1.281162	-0.145909	0.044785
**C**	0.416176	1.514679	0.737322	**O**	-0.613053	-0.644601	0.365753
**O**	1.413177	1.843139	1.371693	**H**	-0.859029	-0.767465	1.294531
**O**	0.072591	1.885568	-0.456636	**C**	-1.702064	0.069825	-0.345407
**H**	1.039530	-0.977231	1.214849	**H**	-1.354888	0.054277	-1.385321
**H**	-0.092197	-2.443111	-0.398203	**C**	-1.802401	1.498431	0.159732
**H**	-2.376904	-2.420192	0.123118	**C**	-2.978054	-0.738633	-0.201491
**H**	-3.346691	-0.689969	1.247422	**H**	-2.834435	-1.765541	-0.557529
**H**	-0.521281	0.661600	2.487475	**H**	-3.311221	-0.765563	0.846974
**H**	-3.527890	-0.157618	-1.310774	**H**	-3.777505	-0.272795	-0.792293
**H**	-1.629566	-1.152603	-2.179803	**H**	-2.099914	1.521212	1.218230
**H**	0.024321	0.324833	-1.093755	**H**	-2.563360	2.040026	-0.417522
**H**	-0.264022	-1.771980	2.914674	**H**	-0.846993	2.026298	0.047938
**Na**	-2.086368	2.250176	-0.701026	***H_2O···IPA-1***			
**C**	4.365650	-0.175608	0.719458	**O**	2.528306	-0.000635	-0.372997
**H**	3.619301	0.405003	1.277650	**H**	3.070842	0.766097	-0.149834
**H**	4.577133	-1.108683	1.259331	**H**	3.072579	-0.767273	-0.153759
**H**	5.296365	0.407880	0.666264	**O**	0.084161	-0.002141	1.222220
**C**	3.853214	-0.483779	-0.683446	**H**	0.886324	-0.002532	0.673077
**H**	4.643835	-1.014887	-1.241652	**C**	-1.051279	0.000382	0.351930
**O**	2.736297	-1.388658	-0.570068	**H**	-1.916391	0.001144	1.031912
**H**	1.999282	-1.059637	-1.104372	**C**	-1.090244	1.270098	-0.499383
**C**	3.468442	0.777609	-1.450673	**H**	-0.231232	1.296715	-1.186776
**H**	4.344519	1.429122	-1.569907	**H**	-1.051234	2.159871	0.142183
**H**	3.100163	0.524588	-2.455333	**H**	-2.009322	1.312279	-1.101858
**H**	2.695739	1.344262	-0.914547	**C**	-1.094231	-1.267730	-0.501559
^{G}NaAlg···ZnO-1				**H**	-2.013456	-1.305980	-1.104061
**O**	-4.451488	0.198577	-0.348960	**H**	-0.235342	-1.295878	-1.189032
**Zn**	-2.937037	0.958958	-0.004460	**H**	-1.057981	-2.158734	0.138438
**C**	1.476130	-1.559852	0.012786	***H_2O···IPA-2***			
**C**	2.955238	-1.200002	-0.139243	**O**	2.736322	0.039300	-0.191355

**C**	3.103452	0.163328	-0.849569	**H**	3.399565	-0.636510	-0.367272
**C**	2.015126	1.152700	-0.345954	**H**	1.923154	-0.448421	0.039992
**O**	1.560952	0.722026	0.969692	**O**	0.163145	-0.961571	0.399466
**C**	0.796397	-0.469941	0.902008	**H**	-0.046925	-0.918744	1.341925
**O**	2.494464	2.438320	-0.244475	**C**	-0.676985	-0.009556	-0.298229
**O**	4.405976	0.693620	-0.608883	**H**	-0.332442	-0.084348	-1.338413
**O**	3.610213	-1.167223	1.133898	**C**	-2.139085	-0.432496	-0.214596
**O**	0.948099	-1.626031	-1.308752	**H**	-2.498624	-0.384347	0.825012
**H**	1.386290	-2.535442	0.510667	**H**	-2.266116	-1.459056	-0.580409
**H**	3.465445	-1.979893	-0.714232	**H**	-2.768325	0.236045	-0.818529
**H**	2.993952	0.038891	-1.932172	**C**	-0.430899	1.405776	0.213253
**H**	1.141779	1.150387	-1.010984	**H**	-1.010936	2.131180	-0.373812
**H**	3.462201	2.364406	-0.167511	**H**	-0.745475	1.496711	1.264783
				**H**	0.632200	1.663316	0.139270

Table S2. Calculated changes in thermodynamic functions for the 1:1 associate formation.

**B3LYP-D3(BJ)/aug-cc-pVDZ**			**ΔH, kJ mol^{-1}**	**ΔG, kJ mol^{-1}**	**ΔH$_{max}$, kJ mol^{-1}**	**ΔG$_{min}$, kJ mol^{-1}**
**^{M}NaAlg**	**ZnO**	1	-321.1	-277.9	-321.1	-277.9
		2	-296.3	-254.2		
		3	-288.5	-238.1		
	**H_2O**	1	-30.6	7.1	-74.7	-37.3
		2	-61.4	-22.9		
		3	-74.7	-37.3		
	**IPA**	1	-85.7	-43.4	-85.7	-43.4
		2	-73.3	-33.7		
		3	-30.9	9.5		
**^{G}NaAlg**	**ZnO**	1	-299.37	-256.1	-299.4	-256.1
		2	-294.508	-248.6		
		3	-205.041	-157.3		
		4	-122.81	-83.1		
		5	-167.877	-120.9		
	**H_2O**	1	-26.6	11.3	-68.5	-34.9
		2	-36.2	1.0		
		3	-68.5	-34.9		
	**IPA**	1	-33.2	10.1	-81.4	-37.8
		2	-46.6	-2.3		
		3	-81.4	-37.8		
**ZnO**	**H_2O**	1	-94.5	-59.7	-94.5	-59.7
	**IPA**	1	-122.2	-83.6	-122.2	-83.6
**H_2O**	**IPA**	1	-15.4	11.9	-22.5	10.4
		2	-22.5	10.4		

Table S3. Distance (d), ratio of d to the sum of the van der Waals radii of the interacting atoms ration of the distance to the sum of VW radiuses, WBI, and FBO.

Associate		Interaction	WBI	FBO	d, Å	Ratio, %
^{G}NaAlg	H_2O	Na//O	0.377	0.479	2.2467	59.3%
		O(COO^-)//H	0.220	0.066	1.8676	68.7%
	IPA	Na//O	0.352	0.382	2.2494	59.4%
		O(COO^-)//H(OH)	0.204	0.065	1.8745	68.9%
		O(CH_2OH)//H(CH_3)	0.050	0.036	2.5364	93.3%
	ZnO	Na//O	0.694	0.658	2.14155	56.5%
		O(COO^-)//Zn	0.845	0.977	1.90446	65.4%
^{M}NaAlg	H_2O	Na//O	0.384	0.489	2.2518	59.4%
		O(COO^-)//H	0.265	0.092	1.7649	64.9%
	IPA	Na//O	0.323	0.419	2.2701	59.9%
		O(COO^-)//H(OH)	0.233	0.084	1.8181	66.8%
		O(COO^-)//H(CH_3)	0.043	0.027	2.6195	96.3%
	ZnO	Na//O	0.732	0.699	2.0935	55.2%
		O(ring)//Zn	0.130	0.157	2.7304	93.8%
		O(COO^-)//Zn	0.941	1.090	1.8688	64.2%
ZnO	H_2O	Zn//O	0.698	0.881	2.0223	69.5%
	IPA	Zn//O	0.699	0.841	1.9849	68.2%
H_2O	IPA	H//O	0.200	0.085	1.8682	68.7%
		O//H(CH_3)	0.032	0.023	2.6769	98.4%

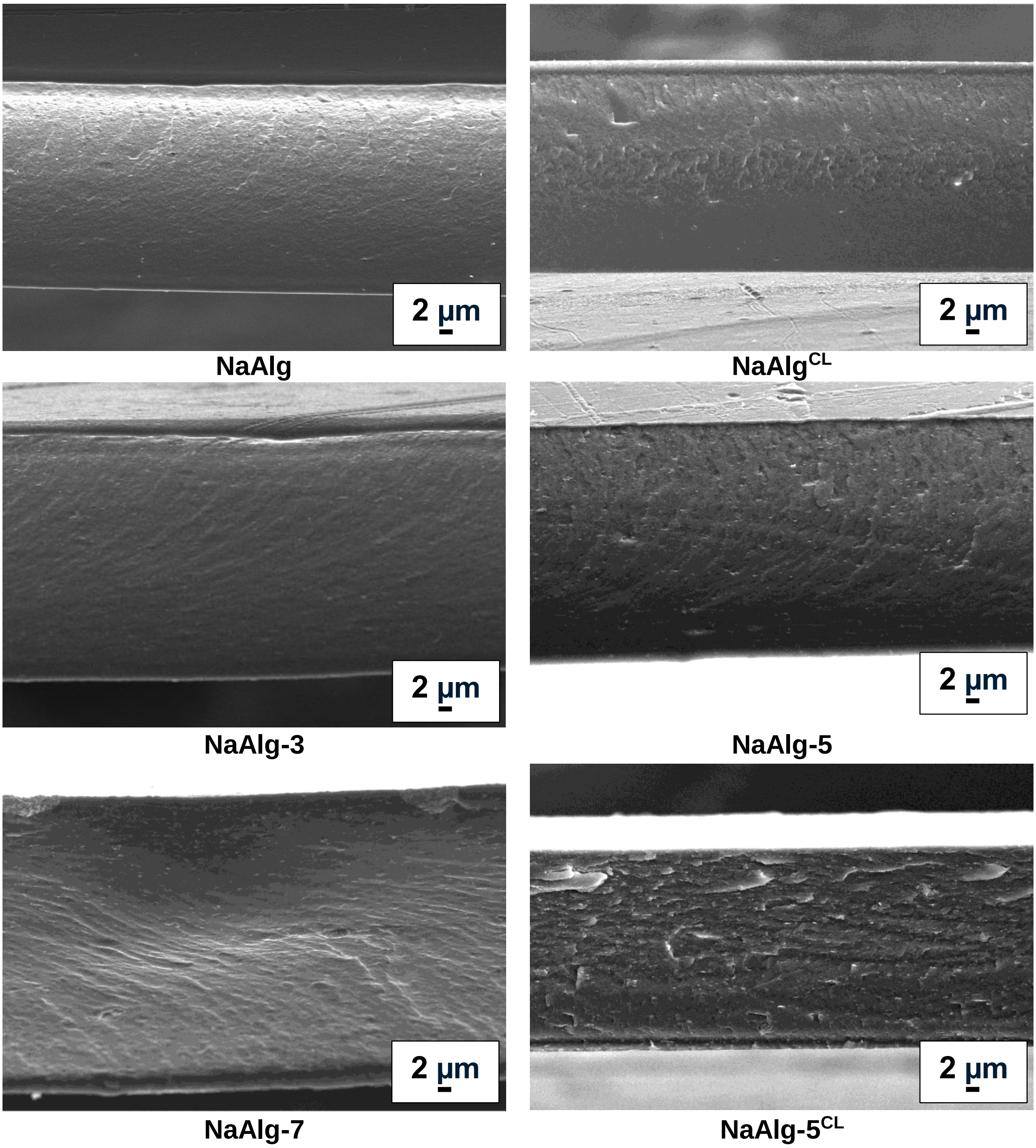

Figure S1. Cross-sectional SEM micrographs of the dense membranes at ×3000 magnification.

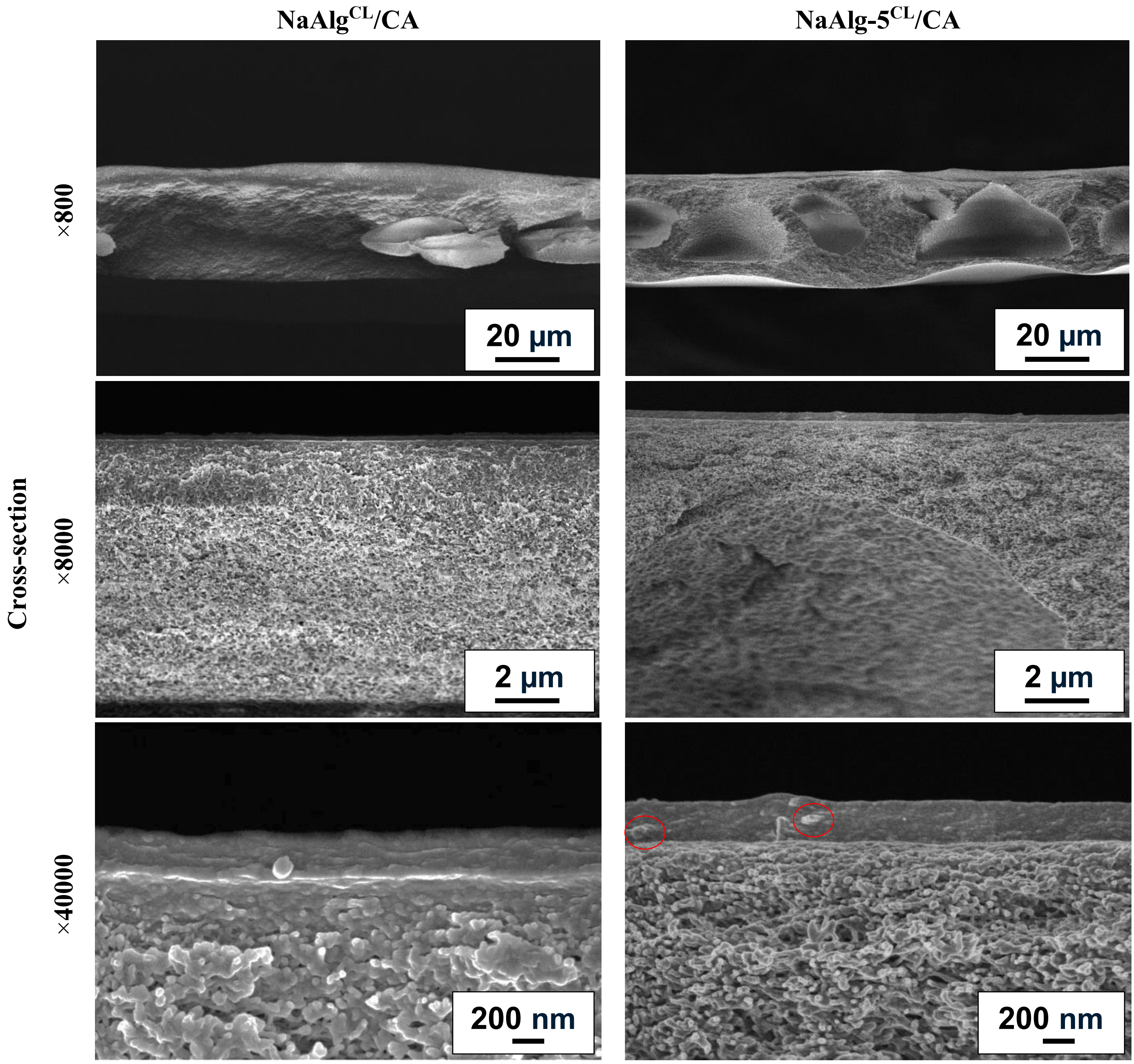

Figure S2. Cross-sectional SEM micrographs of the supported membranes at ×800, ×8000, and ×40,000 magnifications.

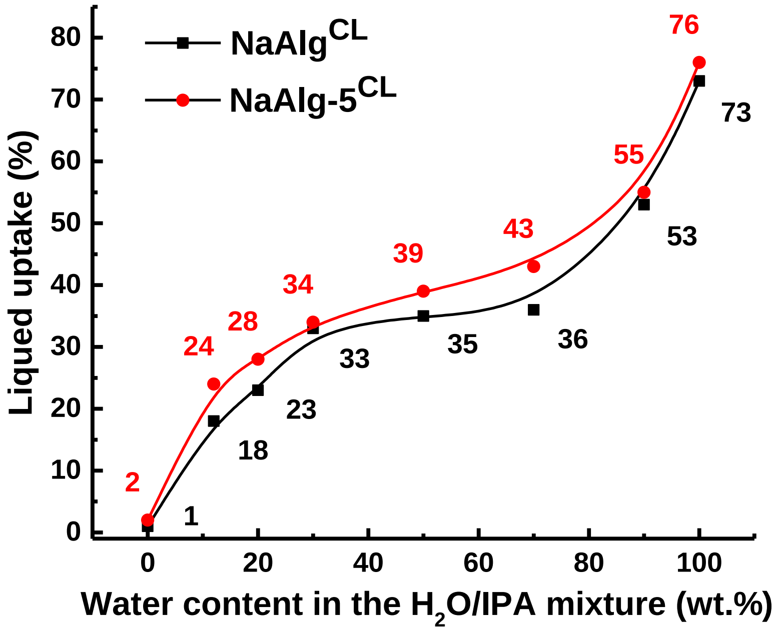

Figure S3. Dependence of liquid uptake on the water content in the H_2O/IPA mixture for cross-linked NaAlg^{CL} and NaAlg-5^{CL} membranes.
